# Frontopolar cortex represents complex features and decision value during choice between environments

**DOI:** 10.1016/j.celrep.2023.112555

**Published:** 2023-05-23

**Authors:** Chun-Kit Law, Nils Kolling, Chetwyn C.H. Chan, Bolton K.H. Chau

**Affiliations:** 1 Department of Rehabilitation Sciences, The Hong Kong Polytechnic University, Hong Kong; 2 University Research Facility in Behavioral and Systems Neuroscience, The Hong Kong Polytechnic University, Hong Kong; 3 Université Lyon 1, INSERM, Stem Cell and Brain Research Institute U1208, 18 Avenue Doyen Lepine, 69500 Bron, France; 4 Department of Psychiatry, University of Oxford, Oxford OX3 7JX, UK; 5 Department of Psychology, The Education University of Hong Kong, Hong Kong

**Keywords:** frontopolar cortex, ventromedial prefrontal cortex, decision making, environment choice, convolutional neural network, CNN

## Abstract

Important decisions often involve choosing between complex environments that define future item encounters. Despite its importance for adaptive behavior and distinct computational challenges, decision-making research primarily focuses on item choice, ignoring environment choice altogether. Here we contrast previously studied item choice in ventromedial prefrontal cortex with lateral frontopolar cortex (FPl) linked to environment choice. Furthermore, we propose a mechanism for how FPl decomposes and represents complex environments during decision making. Specifically, we trained a choice-optimized, brain-naive convolutional neural network (CNN) and compared predicted CNN activation with actual FPl activity. We showed that the high-dimensional FPl activity decomposes environment features to represent the complexity of an environment to make such choice possible. Moreover, FPl functionally connects with posterior cingulate cortex for guiding environment choice. Further probing FPl’s computation revealed a parallel processing mechanism in extracting multiple environment features.

## Highlights

• FPl and vmPFC are involved in environment choice and item choice, respectively
• FPl and a convolutional neural network (CNN) share similar neural representations
• Analyzing the CNN suggests FPl extracts environment features via parallel processing
• FPl functionally connects with dPCC for guiding environment choice

## Graphical abstract

**Figure:**
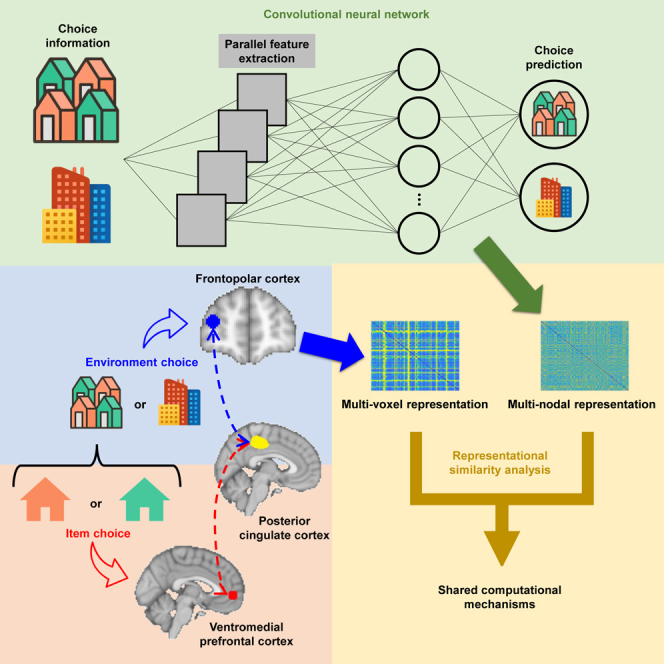

## Teaser

Neuroscientists thought that the ventromedial prefrontal cortex (vmPFC) is involved in signaling all kinds of decisions. Here, Law et al. reveal that the frontopolar cortex (FPC), rather than vmPFC, is involved when people are choosing between complex environments. Artificial intelligence showed that FPC extracts complex choice features via parallel processing.

## Introduction

Big decisions often involve selecting between abstract options that constrain future environments and potential choices within them. For example, when searching for a new house, one may begin by choosing between residential areas or price ranges, pre-selecting or filtering out many a set of potential options in favor of another, before deciding between the actual houses. Here, we refer to such a pre-filtered set of options as an “environment,” which comprises a large number of houses (referred to as “items”).

Selection of an environment (e.g., residential area) has important long-term consequences of determining the concrete items (e.g., houses) the chooser will encounter. In ancient times, a suboptimal environment choice could even be disastrous (e.g., wiping out a whole tribe after migrating to a poor land). Importantly, the computational demands of making environment choices are qualitatively distinct from item choices. Compared with item choice, environment choice involves higher levels of complexity (as the choice information is more multiplex), abstraction (as the choice consequence is about future choice opportunities, instead of actual reward received), and reward prospect (as the choice consequence is more prolonged). In particular, to make more adaptive decisions, retention of complex information is vital in environment choice. A good way to achieve this is through decomposition of the complex information into a few highly informative dimensions. However, despite the related findings on the information selection process in decisions involving numerous choices,^1^^,^^2^^,^^3^ existing decision-making frameworks were mainly developed based on item choice and therefore cannot extend to explain such features unique to environment choice.

We are equally ignorant about the neural mechanisms underlying environment choice, despite its evolutionary relevance, unique computational needs, and distinct behavioral responses. Previous studies trying to identify the brain regions responsible for option evaluation often required participants to choose between concrete items, such as food or probabilistic gambles. Then they computed the overall decision value (DV) to correlate it with brain activity under the assumption that any region evaluating the offers should have scaled activity linked to the decision/comparison signal itself.^4^ However, these approaches generally provide little room to test how the value of a complex option is integrated in the brain.

Applying neural networks can help in solving this problem. First, it provides a data-driven DV estimate that can help identifying a region of interest (ROI) for further analyses. Second, and critically, the high-dimensional architecture of a neural network allows further testing of whether the ROI and neural network share a similar computational mechanism. While there are many neural network architectures, here, convolutional neural networks (CNNs) are of specific interest. This is because CNNs are widely used in computer vision for recognizing objects from complex visual information. Normally, it involves parallel processing for extracting multiplex features from the data, which is particularly useful for studying environment choices that are characterized by multifaceted choice information.

A good candidate region for environment choice in humans is lateral frontopolar cortex (FPl), as it has been implicated in related cognitive processes, such as abstract reasoning, information integration, and prospective memory.^5^^,^^6^^,^^7^ However, it is unclear how exactly FPl might contribute to such decisions. Ventromedial prefrontal cortex (vmPFC) has also been proposed to have a role in decision making, albeit a more general one. It carries essential valuation signals of a wide range of items, such as food and lotteries.^4^^,^^8^^,^^9^^,^^10^^,^^11^^,^^12^^,^^13^^,^^14^ Beyond correlative evidence, lesions of vmPFC are associated with impairments in decision making.^15^^,^^16^ These findings lead to the notion that vmPFC is universally involved in all kinds of choices, namely the “neural common currency hypothesis.”^17^ Recently, however, the universality of vmPFC’s involvement has been challenged.^18^^,^^19^ For example, when people choose between accepting an item and searching in an environment for new items, the classical value comparison signal is absent in vmPFC. Since the natures of environments and items are fundamentally different, at least in terms of their levels of abstraction and reward prospect, it is unclear whether vmPFC’s role in decision making is generalizable to environment choice.

Importantly, any brain region involved in environment choice should pass the following three tests. First, for making comparisons between environments, the region should contain a “value comparison signal,” a signal that scales with the difference in value between environments. Second, the region’s value comparison signals should be sensitive to contextual factors that alter choices. Third, the signal should be linked to value itself, instead of confounding factors such as salience of the environments.

In this study, we designed a two-stage decision-making task to investigate the neural mechanisms underlying environment choice using functional magnetic resonance imaging (fMRI). Stage 2 of the task was a typical decision-making task that involved choosing between two items. The preceding stage 1 involved an environment choice that determined items that were subsequently available in stage 2 for selection. To extract environmental features and overall DV, we trained a behaviorally optimized and brain-naive CNN. In a series of univariate analyses and representational similarity analyses (RSA) comparing the model elements with neural activity, we observed dissociable patterns of activity in FPl and vmPFC in environment choice and item choice. In addition, we showed that the computation of FPl is related the extraction of environment information and the computation of dorsal posterior cingulate cortex (dPCC) is related to the combination of such information for guiding choices. This functional network between FPl and dPCC was also confirmed in a psychophysiological interaction (PPI) analysis. Further probing FPl’s computation revealed a more specific mechanism of parallel processing in extracting multiple environment features for decision making. These results suggest FPl extracts multiple choice features and passes information to dPCC for guiding environment choice.

## Results

### Behavioral results

**Figure 1:**
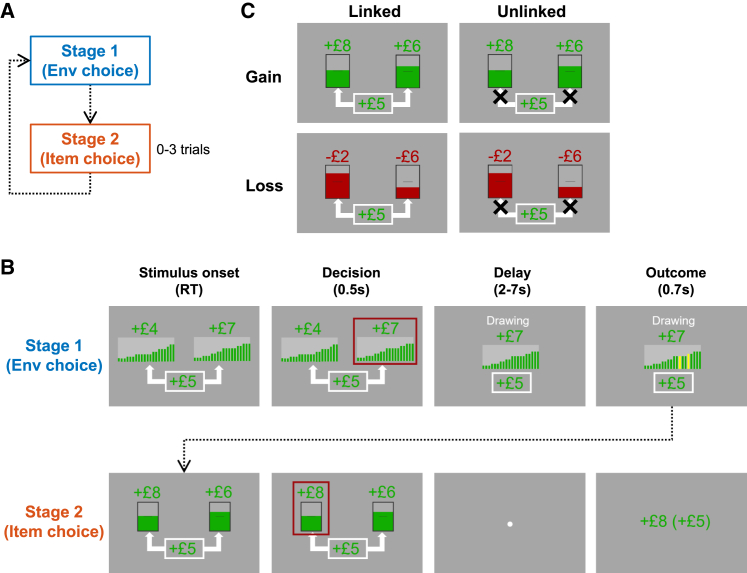
A two-stage decision-making task (A) Each block began by a stage 1 (environment choice) trial and followed by zero to three stage 2 (item choice) trials. (B) On each stage 1 trial, participants chose between two environments. Each environment consisted of 20 items, with 20 bars representing their reward probabilities and a number representing their average reward magnitude (stimulus onset). After a decision was made, the chosen environment was surrounded by a red frame (decision) and presented at the center (delay). After a delay, two items were drawn pseudo-randomly and highlighted in yellow (outcome) and offered on the subsequent stage 2 item choice trial (stimulus onset). After a decision, the chosen item was surrounded by a red frame (decision). A fixation dot appeared (delay) and after a delay the amount of probabilistic reward earned from the chosen item was presented (outcome). (C) Two bonus conditions were introduced to test whether decision signals were adaptive to context. In the linked bonus condition (left column; indicated by two arrows), the bonus was delivered only if the probabilistic reward of the chosen item on the same stage 2 trial was won. In unlinked bonus condition (right column; indicated by crosses), the bonus was delivered unconditionally on each stage 2 trial. To distinguish neural signals pertinent to DV or valence, each block was also assigned to one of the three gain/loss conditions. The two options were either related to gains (top row), losses (bottom row), or a combination of gains and losses. The figure shows examples of stage 2 trials, while stage 1 trials involved the same color codes for indicating gain/loss conditions and the same symbols for indicating bonus conditions. See also Figure S1.

To examine the neural mechanisms of environment choice, we developed a two-stage decision-making task (Figures 1A and 1B). Each block of trials included one environment choice trial (stage 1), followed by zero to three item choice trials (stage 2). Item choice trials were similar to a typical binary decision-making task, which required participants to choose between two items associated with different reward magnitudes and probabilities. On environment choice trials, participants chose between two environments, each of which was an aggregate of 20 items. Then, on each subsequent item choice trial, two items were pseudo-randomly drawn from a chosen environment for selection. As such, the stage 1 environment choice determined the items that were made available subsequently, as opposed to stage 2 item choice, which directly determined the probabilistic reward. Hence, selecting an advantageous environment provides a prospect of generally more rewarding items in the same block of trials.

In addition, to test whether the neural decision signals were flexibly modulated under different contexts, we introduced two different bonus conditions (linked vs. unlinked condition; Figures 1B, 1C, and S1) that required participants to either regulate or ignore the choices’ reward probabilities. In the linked condition, when a gamble worked out from the chosen item, the bonus was delivered in addition to the reward of the chosen item itself. Hence, when the bonus is more rewarding, participants ought to be more sensitive to the item’s reward probability. The unlinked condition served as a control, as the bonus was delivered to participants regardless of the outcome of the chosen item. Participants’ choices should thus be unaffected by the presence of the bonus. Besides, neural signals related to choice value are easily confounded by choice salience when experiments only involve options that are rewarding.^4^^,^^20^ To test the value-based neural decision signals, this experiment also included options (both environments and items) associated with loss to orthogonalize choice value and choice salience (Figure 1C). These manipulations are important to discern the neural decision signals and are detailed further in later sections.

**Figure 2:**
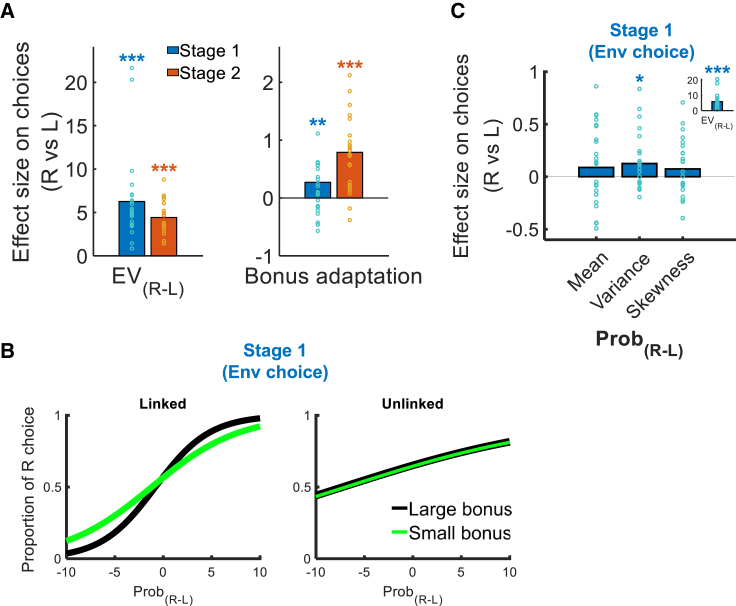
Behavioral results (A) A logistic regression showed positive effects of EV_(R-L)_ and bonus adaptation (i.e., a Prob_(R-L)_ × Bon × Cond three-way interaction) on participants’ choices in both stage 1 environment choice and stage 2 item choice. (B) Psychometric curves that illustrate the bonus adaptation in environment choice. In linked condition, when the bonus value became larger, there was a stronger preference to choose environments with larger reward probabilities (left panel). In contrast, in unlinked condition, the preference for larger reward probabilities was not affected by the bonus value (right panel). (C) In addition to environments with larger EVs (inset), participants preferred those with larger variances (main panel). EV_(R-L)_, difference in expected value; Prob_(R-L)_, difference in reward probability; Bon, bonus value; Cond, bonus condition; mean_(R-L)_, Variance_(R-L)_, Skewness_(R-L)_, differences in mean, variance, and skewness of reward probability between environments respectively. ^∗∗∗^p < 0.001, ^∗∗^p < 0.01, and ^∗^p < 0.05 (n = 24; one-sample t test). Data points represent individual participants. See also Figures S2 and S3.

A general linear model (GLM1; see STAR Methods section, “behavioral analysis”) was applied to test whether participants (1) chose options that were better in expected values (EVs), and (2) chose according to the bonus context. Not surprisingly, when the EV of the rightward environment was larger (i.e., large EV_(R-L)_), participants were more likely to choose the rightward environment (*β* = 6.264, *t*_23_ = 6.196, p = 2.543 × 10^−6^; Figures 2A, S2A, and S2C; Table S1). Importantly, participants also chose according to the context by showing a bonus adaptation (i.e., a Prob_(R-L)_ × Bon × Cond three-way interaction: *β* = 0.271, *t*_23_ = 3.065, p = 0.006; Figures 2A, 2B, S2A, and S2C; Table S1). When the winning of bonus was associated with item probability (i.e., linked condition), participants adjusted flexibly to prefer environments with large reward probability when the bonus was large. As such, they were more likely to encounter items with large reward probabilities to earn the bonus subsequently.

A similar GLM was applied to analyze the item choice data. Akin to the environment choice results, items with lager EVs were preferred (*β* = 4.420, *t*_23_ = 11.582, p = 4.468 × 10^−11^; Figures 2A, S2A, and S2C; Table S1) and there was also a presence of bonus adaptation (*β* = 0.790, *t*_23_ = 4.735, p = 9.007 × 10^−5^; Figures 2A and S2A–S2C; Table S1). Taken together, in both environment and item choices, participants did choose options with larger EVs and adjust their choices according to the bonus condition. These results also showed that participants understood that the offers in item choice were determined by the environments they selected earlier.

For environment choice, it is critical to integrate information from the component items of each environment to form representations of statistical moments (e.g., the mean, variance, skewness of the item distribution) to guide decisions. We then carried out GLM2 (see STAR Methods section, “behavioral analysis”) to test whether participants considered any statistical moments during environment choice. The results showed an absence of effect of mean on environment choice (*β* = 0.089, *t*_23_ = 1.121, p = 0.274; Figure 2C). This was because the component items could lead to either gains or losses; the preferences for mean probability were opposite under gains and losses such that the effects of mean probability in these two conditions were canceled out (Figure S2D). Besides, we found that environments with larger variance (i.e., greater diversity) were preferred (*β* = 0.126, *t*_23_ = 2.248, p = 0.035; Figure 2C). Choosing environments with greater variance was advantageous because participants could encounter more variable items on the subsequent stage 2 trials, where they could then accept the more rewarding items and reject the poor items. Finally, no effect of skewness on choices was observed (*β* = 0.074, *t*_23_ = 1.290, p = 0.210). An additional analysis showed that the preference for larger variance was not confounded by any exploratory behaviors due to unfamiliarity of the task (Figure S2E). To confirm the reproducibility and generalizability of the results of the statistical moment preferences, we carried out another behavioral experiment that involved a simplified environment choice task that was similar to the stage 1 of the main experiment (Figure S3A). Consistently, we found that environments with larger variance were preferred (Figure S3B).

### CNN best predicts choice behavior

**Figure 3:**
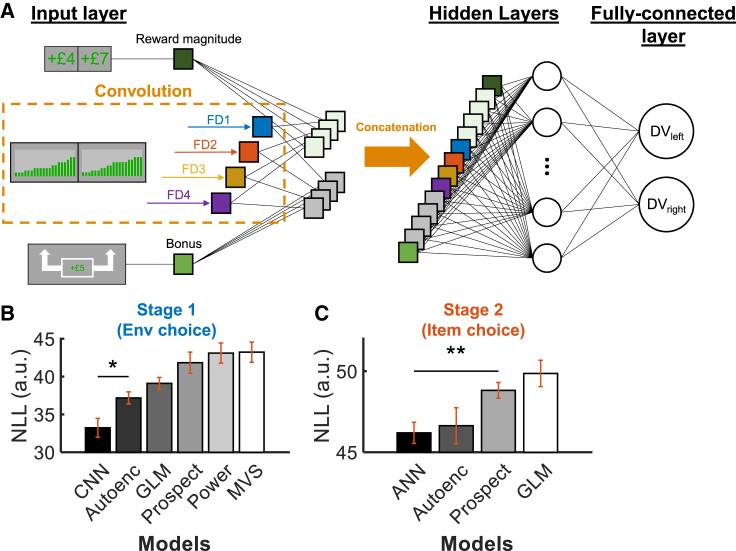
A CNN for predicting environment choice behavior (A) A simplified schematic of the CNN model. The CNN receives value of each environment as the input. Reward probability in the input is convolved by four feature detectors to form four sets of feature maps. Next, the feature maps are multiplied by the reward magnitude and bonus information and concatenated a series of hidden layers for information integration. Finally, in the fully connected layer, information is combined to form DVs for the leftward or rightward environments. (B) Model comparison results revealed the CNN outperforms other alternative models in predicting participants’ environment choices. (C) A similar artificial neural network (ANN) also best predicts item choices. NLL, negative log likelihood; Autoenc, autoencoder; Power, power law model; Prospect, cumulative prospect theory. ^∗∗^p < 0.01, ^∗^p < 0.05 (n = 20; independent samples t test). Error bars represent means ± SEM. See also Figure S4.

So far, applying GLM1 held an assumption that participants perfectly estimated the EVs of the options and made their choices accordingly. However, each environment consisted of information from 20 items, and it is unclear how the integration of this complex information occurred computationally. Similar to environment choice, visual object recognition also involves integrating complex information from the retina. In artificial intelligence, the CNN is successful in computer vision not only because it can decompose complex visual information and perform nonlinear integration to decode image identity but also because it resembles the hierarchical neural process of the human ventral visual pathway.^21^^,^^22^ Hence, we adapted a CNN for analyzing complex decision information to decode environment choices (see STAR Methods section, “stage 1 CNN for environment choice” for details). In brief, environment information is fed in as the input of the CNN (Figures 3A and S4Ai). The component items of the environments are convolved by four different feature detectors and transformed into four sets of feature maps. All feature maps were then integrated into a DV for each environment for choice prediction.

We tested carefully different CNN architectures before analyzing the fitted data further (see Figure S4Aii for details). The best CNN architecture (prediction accuracy = 87%; Figure S4Aiii) involves 512 nodes and four feature detectors in the size of 2 × 1. Next, we compared the performance of the CNN with other alternative models (see STAR Methods section, “computational modeling”). The results showed that the CNN significantly outperforms other models (*t*s_38_ < −2.661, *P*s < 0.012; Figure 3B). We found similar results when we applied the same models to fit the data of the simplified behavioral experiment: the CNN best fits the environment choices (Figure S3C).

The same set of models was applied to find the model that best describes participants’ item choices for subsequent neural analyses (except the mean-variance-skewness [MVS] model and power law model, which are not applicable). Notably, the input for item choices only contains values of two items, as opposed to that for environment choices, which involves information of 40 items. Convolution is not applicable for item choice, and the CNN for environment choice was reduced to an artificial neural network (ANN) by the absence of a convolution (Figure S4B). Interestingly, a model comparison revealed the ANN also outperforms other alternative models in predicting item choices (*t*s_38_ < −3.226, *P*s < 0.003; Figure 3C), except the autoencoder (*t*_38_ = −0.345, p = 0.732).

### Complex environment values were encoded in FPl

**Figure 4:**
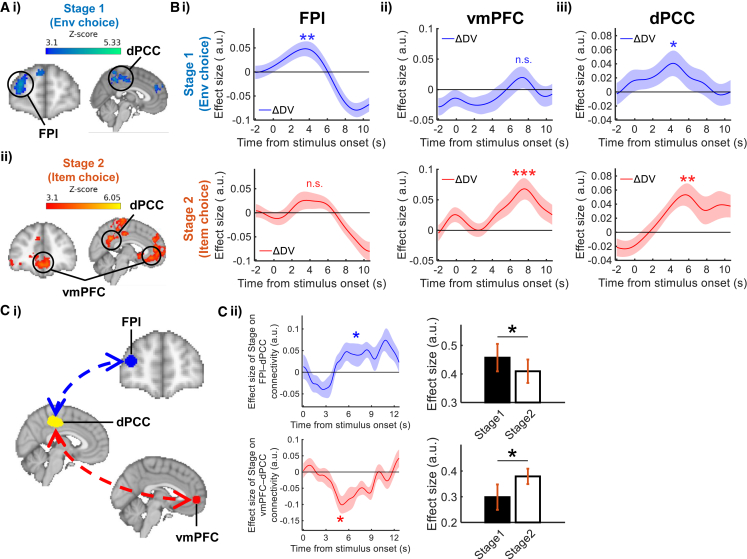
Dissociable roles of FPl and vmPFC in environment choice and item choice (A) A whole-brain analysis identified (i) FPl signal was related to the difference in DV (ΔDV) between environments in stage 1 and (ii) vmPFC signal was related to the ΔDV between items in stage 2. Dorsal posterior cingulate cortex (dPCC) showed both ΔDV signals in stages 1 and 2. (B) Time courses of the ΔDV signals in (i) FPl, (ii) vmPFC, and (iii) dPCC in environment choice and item choice. (C) (i) A psychophysiological interaction (PPI) analysis was conducted among dPCC, FPl, and vmPFC. (ii) The functional connectivity between FPl and dPCC was stronger during stage 1 environment choice than stage 2 item choice (top row), whereas the functional connectivity between vmPFC and dPCC showed the opposite pattern (bottom row). ^∗∗∗^p < 0.001, ^∗∗^p < 0.01, and ^∗^p < 0.05 (n = 24; one-sample t test). Shading represent mean ± SEM. See also Figure S5 and Table S2.

One key feature of the CNN is that it integrates complex choice information into a single DV for guiding decisions. Previous studies showed that, during item choice, the option DV was strongly related to vmPFC activity.^10^^,^^23^^,^^24^^,^^25^^,^^26^ Hence, we used this CNN-derived DV to scrutinize whether environment and item choices involved the same vmPFC region or dissociable neural mechanisms. We analyzed the whole-brain fMRI data by applying GLM3, which included the DV difference between environments (i.e., ΔDV_Env_) and the DV difference between items (i.e., ΔDV_Item_). Interestingly, a dissociation in the DV difference signals of the two stages was observed. First, we replicated a widely reported finding that vmPFC activity correlated with ΔDV_Item_ in item choice (Montreal Neurological Institute [MNI]= [2, 40, −10], cluster-based thresholding *Z* > 3.1, p = 7.77 × 10^−21^; Figure 4ii). Surprisingly, when we investigated environment choice, there was an absence of ΔDV_Env_ signal in vmPFC. Instead, FPl activity was found positively correlated with ΔDV_Env_ during environment choice (MNI = [38, 52, 22], cluster-based thresholding *Z* > 3.1, p = 1.19 × 10^−7^; Figure 4Ai) but not correlated with ΔDV_Item_ during item choice. This is supported by a contrast that directly compared the ΔDV_Env_ and ΔDV_Item_ effects: the ΔDV_Env_ signal was stronger than the ΔDV_Item_ “signal” only in FPl (Figure S5A). Other regions that showed a significant ΔDV_Env_ or ΔDV_Item_ signal are reported in Table S2. Similar results were also yielded if the CNN-derived DVs were replaced by a conventional EV (i.e., reward magnitude multiplied by reward probability) term as regressors, and the results were consistent with previous findings (Figures S5B and S5C).

Despite the double dissociation of FPl and vmPFC in environment choice and item choice, it is equally important to ascertain that the absence of signal in FPl and vmPFC was not merely due to the conservative statistical corrections in the whole-brain analysis. Hence, an ROI analysis was performed by placing a mask over these regions and testing their signal time courses. The results were broadly consistent with those from the whole-brain analysis. In environment choice, the ΔDV_Env_ signal ramped up in FPl and peaked at 3.507 s after the environments were presented (*β* = 0.046, *t*_23_ = 3.032, p = 0.006, BF_10_ = 7.526; Figure 4Bi, top panel), while no significant ΔDV_Item_ signal was observed in item choice (*β* = 0.023, *t*_23_ = 1.409, p = 0.172, BF_10_ = 0.514; Figure 4Bi, bottom panel). In contrast, in vmPFC, there was a ΔDV_Item_ signal in item choice that peaked at 7.813s (*β* = 0.067, *t*_23_ = 3.806, p = 9.105 × 10^−4^, BF_10_ = 38.074; Figure 4Bii, bottom panel) but an absence of ΔDV_Env_ signal in environment choice (*β* = 0.018, *t*_23_ = 1.011, p = 0.323, BF_10_ = 0.339; Figure 4Bii, top panel). The double dissociation was also supported by the overall deactivation patterns of FPl and vmPFC (Figure S5D) and a two-way ANOVA (two regions times two stages) that compared the sizes of ΔDV_Env_ and ΔDV_Item_ signals of FPl and vmPFC (interaction term: *F*(1,23) = 16.026, p = 0.001). Taken together, the findings provided further evidence that, in FPl, a DV difference signal was only present during environment choice and was absent during item choice, whereas the vmPFC showed the opposite pattern.

Finally, we inspected the signals in dorsal posterior cingulate cortex (dPCC), which exhibits DV signals during item choice and foraging.^4^^,^^27^^,^^28^^,^^29^ In the whole-brain analysis, dPCC showed a positive ΔDV signal in both environment choice (MNI = [16, 32, −42], cluster-based thresholding *Z* > 3.1, p = 2.44 × 10^−13^; Figure 4Ai) and item choice (MNI = [8, −52, 36], cluster-based thresholding *Z* > 3.1, p = 1.73 × 10^−24^; Figure 4Aii). ROI analysis also revealed that dPCC activity was correlated with ΔDV_Env_ in environment choice (*β* = 0.039, *t*_23_ = 2.199, p = 0.038, BF_10_ = 1.609; Figure 4Biii, top panel) and ΔDV_Item_ in item choice (*β* = 0.051, *t*_23_ = 3.363, p = 0.003, BF_10_ = 14.818; Figure 4Biii, bottom panel).

So far, we observed that dPCC was co-activated with FPl and vmPFC in environment choice and item choice respectively. Next, we tested the functional coupling among these regions using a PPI analysis (Figure 4Ci). The results showed that the functional connectivity between FPl and dPCC was stronger during stage 1 environment choice (i.e., a positive effect; *β* = 0.048, *t*_23_ = 2.529, p = 0.019, BF_10_ = 2.869; stage 1 connectivity = 0.457; stage 2 connectivity = 0.410; Figure 4Cii, top row). In contrast, the functional connectivity between vmPFC and dPCC was stronger during stage 2 item choice (i.e., a negative effect; *β* = −0.081, *t*_23_ = −2.388, p = 0.026, BF_10_ = 2.227; stage 1 connectivity = 0.298; stage 2 connectivity = 0.379; Figure 4Cii, bottom row). Taken together, these results suggest that dPCC connected flexibly to the relevant brain regions (i.e., FPl vs. vmPFC) according to the context of the decision (i.e., environment choice vs. item choice).

### FPl activity reflects decision signals reliably but not value-neutral environment features

Our neural analyses so far suggest that FPl has a specific role in environment choice, whereas vmPFC has a specific role in item choice. To confirm the signals were not confounded by other factors, FPl ΔDV_Env_ and vmPFC ΔDV_Item_ signals must possess at least two additional properties: (1) it should be flexibly modulated only by choice-relevant information (i.e., whether it changed as a function of the bonus only in the linked, but not the unlinked, condition); (2) it should be orthogonal to the option (environment or item) salience. We report two sets of analyses below that demonstrated FPl ΔDV_Env_ signal and vmPFC ΔDV_Item_ signal possessed these two properties.

**Figure 5:**
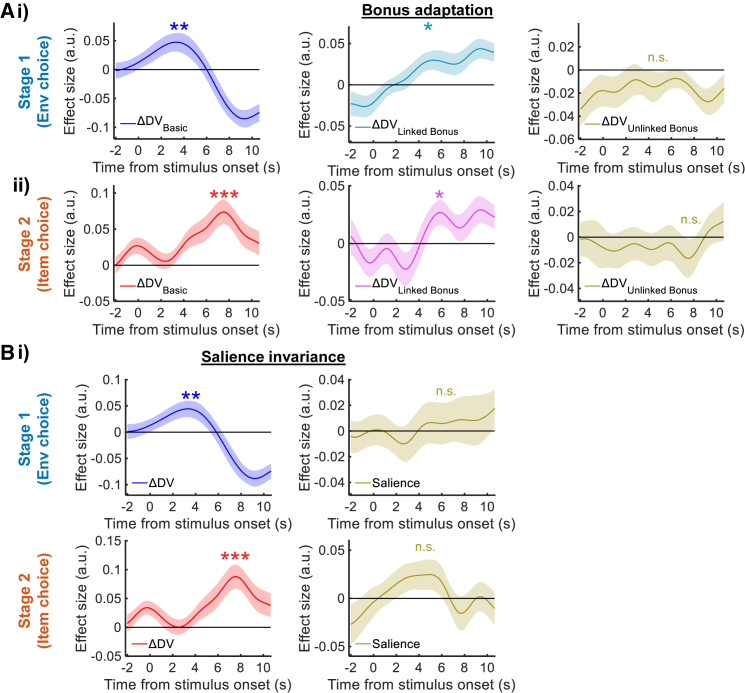
Properties of the ΔDV signals (A) The ΔDV signals adapted flexibly to the bonus. The ΔDV term was split into three components: the ΔDV that excluded the bonus (ΔDV_Basic_), the rest of the ΔDV that was contributed by the bonus in linked condition (ΔDV_Linked Bonus_), and that in unlinked condition (ΔDV_Unlinked Bonus_). (i) In stage 1 environment choice, FPl signal was only modulated by the choice-relevant ΔDV_Linked Bonus_ term (middle panel), but not by the choice-irrelevant ΔDV_Unlinked Bonus_ term (right panel). (ii) vmPFC signal showed significant ΔDV_Linked Bonus_ effect on the first item choice trial and an absence of ΔDV_Unlinked Bonus_ effect. (B) (i) ΔDV signals of FPl in stage 1 and (ii) that of vmPFC in stage 2 were both not confounded by the option salience. ^∗∗∗^p < 0.001, ^∗∗^p < 0.01, and ^∗^p < 0.05 (n = 24; one-sample t test). Shading represents mean ± SEM.

As participants flexibly adapted their choice according to the bonus conditions (Figure 2A), we tested whether FPl and vmPFC signals were also adapted in the same way. To this end, in GLM4 we split the DV into three terms: a basic DV term that concerns all choice information except the bonus (DV_Basic_) and two DV terms that only concerns the bonus value in the linked condition (DV_Linked Bonus_) or in the unlinked condition (DV_Unlinked Bonus_). The DV_Unlinked Bonus_ term served as a control to test that the neural signals were not modulated when the bonus was irrelevant to the choice. Not surprisingly, during environment choice, FPl encoded the ΔDV_Basic_ (*β* = 0.047, *t*_23_ = 2.967, p = 0.007, BF_10_ = 6.621; Figure 5Ai, left panel). In the linked condition, as the selected environment would have an impact on the bonus acquisition in the subsequent item choices, FPl also encoded the bonus value accordingly (*β* = 0.028, *t*_23_ = 2.372, p = 0.026, BF_10_ = 2.167; Figure 5Ai, middle panel). In contrast, in the unlinked condition, the bonus was bound to be delivered (i.e., bonus acquisition was independent of the choices made). Thus, FPl did not signal the ΔDV_Unlinked Bonus_ (*β* = −0.013, *t*_23_ = −0.975, p = 0.340, BF_10_ = 0.329; Figure 5Ai, right panel). These suggest FPl was capable of integrating task-relevant information for environment choice. Likewise, when vmPFC activity in item choice was inspected, there was a ΔDV_Basic_ signal (*β* = 0.072, *t*_23_ = 4.228, p = 3.188 × 10^−4^, BF_10_ = 96.140; Figure 5Aii, left panel) and an absence of ΔDV_Unlinked Bonus_ signal (*β* = −0.015, *t*_23_ = −0.987, p = 0.334; BF_10_ = 0.332; Figure 5Aii, right panel). A ΔDV_Linked Bonus_ signal was present in vmPFC only on the first item choice trial of each block (*β* = 0.025, *t*_23_ = 2.293, p = 0.031, BF_10_ = 1.887; Figure 5Aii, middle panel; see GLM5 in STAR Methods section, “ROI analysis”). If the subsequent item choice trials were included, the ΔDV_Linked Bonus_ signal was no longer significant (*β* = 0.009, *t*_23_ = 0.619, p = 0.542; BF_10_ = 0.255). This was possibly due to the repetition suppression of the bonus signal when the bonus remained the same on subsequent item choice trials.^30^

It is worth noting that we controlled for the potential confounding of salience in our study, because option value and option salience are collinear in many studies. To avoid this we included gain and loss trials to orthogonalize option value and salience. Also, in an ROI analysis, we tested the ΔDV signal by additionally including a salience term in GLM6. It reproduced the result that FPl activity correlated with ΔDV_Env_ even though the factor of salience was included (*β* = 0.043, *t*_23_ = 2.829, p = 0.010, BF_10_ = 5.051; Figure 5Bi, left panel). Crucially, FPl activity was not related to salience (*β* = 0.006, *t*_23_ = 0.343, p = 0.735, BF_10_ = 0.226; Figure 5Bi, right panel). Likewise, when we tested vmPFC signal by the same analysis, vmPFC showed activity correlated with ΔDV_Item_ (*β* = 0.085, *t*_23_ = 4.158, p = 3.799 × 10^−4^, BF_10_ = 82.311; Figure 5Bii, left panel) and it was unrelated to salience (*β* = 0.021, *t*_23_ = 1.456, p = 0.159, BF_10_ = 0.544; Figure 5Bii, right panel).

### Statistical moments of environments are represented in the CNN

**Figure 6:**
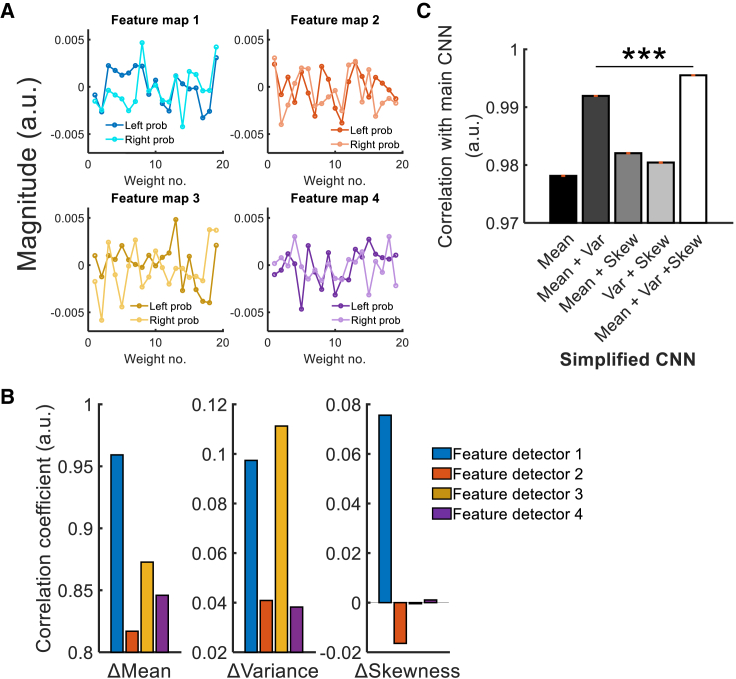
The CNN contains information about statistical moments of environments (A) All feature maps are weigh summed during the computation of DVs. (B) Since the components of the feature maps are weighted differently, the DVs derived from the feature maps also show different degrees of correlation to the differences in mean, variance, and skewness of environments. (C) A representational similarity analysis (RSA) suggested that the CNN carries information about the mean, variance, and skewness of the environments. This was achieved by comparing the representations of the original CNN with different simplified CNNs that receive the actual mean, variance, and/or skewness of the environments directly as input. ^∗∗∗^p < 0.001 (n = 24; signed-rank test). Error bars represent mean ± SEM.

So far, we found that FPl is important to environment choice by showing a signal correlated with the CNN-derived DV parameter. Behavioral analysis results also showed that environment choices were partially guided by statistical moments of the environments’ item distribution (Figure 2C). However, it is unclear whether the statistical moments were represented in FPl and the CNN. In the CNN, the complex information of the environments is decomposed into four sets of feature map. These feature maps contain different patterns of weights on estimating the DV (Figure 6A). Hence, they may carry different information about the environments’ statistical moments. Figure 6A shows the weights of each feature map on estimating the DV. To test whether this is the case, we isolated the feature information extracted by each feature map to obtain a “partial DV” per feature detector. We then correlated each partial DV with the statistical moments (see STAR Methods section, “partial DV contributed by each feature map” for details). All feature maps’ partial DVs are significantly correlated with the differences in mean (*r*s > 0.816, *P*s < 0.001; Figure 6B) and variance (*r*s > 0.038, *P*s < 0.009) between environments but only the partial DV of detector 1 is correlated with difference in skewness (*r* = 0.076, p = 1.658 × 10^−7^). Interestingly, the correlation patterns varied across feature detectors. For example, detector 1 is more strongly related to mean difference compared with those of other detectors (*z*s > 28.908, *P*s < 0.001), while detector 3 is more related to variance difference (comparison with detectors 2 and 4, *z*s > 3.460, *P*s < 5.400 × 10^−4^; comparison with detector 1, *z* = 0.685, p = 0.494). In short, the differential correlation patterns associated with the feature detectors enable the CNN to extract information from the item distributions during environment choice.

To find out whether the CNN does make use of statistical moments to guide environment choice, we tested the representational similarity of the CNN with five “simplified” CNN models that are trained with explicit information about statistical moments. Instead of receiving 20 component items of each environment as input, these simplified models receive either the item mean (mean model), the item mean and variance (mean + Var model), item mean and skewness (mean + skew model), item variance and skewness (Var + skew model), or item mean, variance, and skewness (mean + Var + skew model). Supposedly, the original CNN resembles the fifth model the most, and that was exactly what we found in an RSA. There were significant correlations in the RSAs between the original CNN and these five simplified CNN models (*rho*s > 0.978; zs > 4.413, signed-rank *P*s < 1.017 × 10^−5^, permutation *P*s < 0.001; Figure 6C) and, importantly, the correlation was strongest with the mean + Var + skew model (*rho* = 0.996; zs > 4.413, signed-rank *P*s < 1.017 × 10^−5^, permutation *P*s < 0.001). These suggest the CNN for environment choice is most similar to a model that receives input of the mean, variance, and skewness of the environments.

### The CNN and FPl share similar computational mechanisms during environment choice

So far, we have shown that FPl signal is related to the CNN-derived DV. However, it is unclear whether FPl and CNN also share similar underlying computational processes. Previous studies showed that FPl has an important role of maintaining multiple goals simultaneously,^7^ and these goals can be maintained via a multivariate code.^31^ Likewise, the CNN employs parallel feature detectors to extract multifaceted information from the same environment and represents them via multiple nodes. Hence, we compared whether the CNN and FPl shared similar computational representations while extracting environment information.

**Figure 7:**
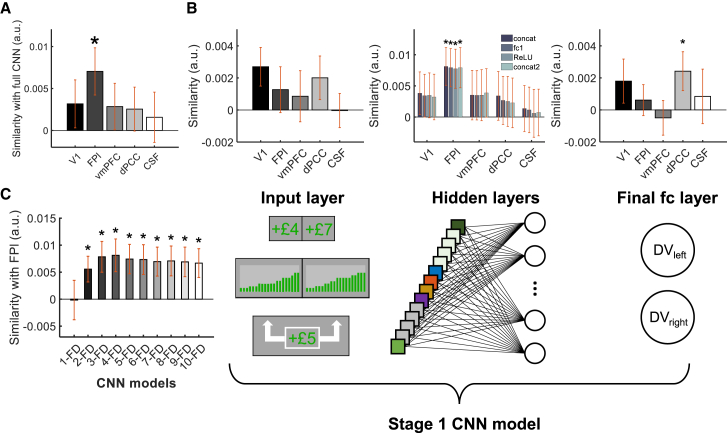
FPl and CNN shared similar neural representations (A) An RSA showed that the multi-nodal activation of the CNN was similar to the multi-voxel activation pattern of FPl but not other control regions. (B) Further RSAs testing individual CNN layers suggested that FPl was specifically related to the hidden layers (extracts and integrates environment features), whereas V1 and dPCC were related to the input layer (receives environment information) and final fc layer (combines environment features into DVs) respectively. (C) FPl was compared with variants of CNNs that involve 1–10 feature detectors. FPl was similar to all variants with multiple detectors but not the CNN that contains only one detector. ^∗^p < 0.05 (n = 24; signed-rank test). Error bars represent mean ± SEM. See also Figure S6.

This comparison was conducted using an RSA that involves two steps. The logic behind an RSA analysis was that we wanted to test whether FPl used the same complex code to represent environments as the CNN trained on human choices (i.e., had the same representational similarity). To begin with, we compared the FPl activity with the full CNN (i.e., concatenating all layers of the CNN). First, we calculated the dissimilarity in multi-voxel activity of FPl (or the multi-nodal activation of the CNN) between each pair of trials in the form of representational dissimilarity matrix (RDM). Second, we tested the similarity in RDMs between FPl and CNN via a Spearman correlation. Note that, during the RSA, the average signal of a brain region is essentially removed, such that the RSA should be orthogonal to the univariate analysis in Figures 4 and 5. Our RSA showed that FPl and CNN showed similar representations (*rho* = 0.007, signed-rank p = 0.016, permutation p = 0.014; Figure 7A). Similar RSAs were applied to vmPFC and dPCC (both are also involved in decision making but not specifically in environment choice), primary visual cortex (V1), and cerebrospinal fluid (CSF; both are unrelated to decision making). No significant correlation was found in these regions (*rho*s < 0.004, signed-rank *P*s > 0.345, permutation *P*s > 0.123; Figure 7A). These results suggest the CNN only shared similar computational mechanism with FPl. Additional RSAs also showed that FPl was only similar to the CNN that predicts environment choice but not to the ANN that predicts item choice. In contrast, vmPFC was similar to the item choice ANN but not the environment choice CNN (Figure S6).

As the CNN contains a multi-layer architecture, we further scrutinized whether the computations of FPl was specifically similar to individual layers. First, an RSA that focused on the input layer only showed a marginally significant representational similarity with V1 (*rho* = 0.003, signed-rank p = 0.056, permutation p = 0.040; Figure 7B, left column), possibly because the raw value input is correlated with sensory information. Second, when the RSA was focused on the subsequent hidden layers where environment information is extracted and integrated, all the hidden layers were reliably correlated with FPl (*rho*s > 0.007, signed-rank *P*s < 0.035, permutation *P*s < 0.024; Figure 7B, middle column) but not with any other regions (*rho*s < 0.004, signed-rank *P*s > 0.317, permutation *P*s > 0.139). Intriguingly, the fully connected layer (which combines choice information into DVs) was correlated with dPCC only (*rho* = 0.002, signed-rank p = 0.022, permutation p = 0.036; Figure 7B, right column) but not with other regions including FPl (*rho*s < 0.002, signed-rank *P*s > 0.492, permutation *P*s > 0.081). On top of the functional coupling between FPl and dPCC (Figure 4C), the RSA results here showed that FPl and dPCC played distinct roles in environment choice. FPl’s computation processes were related to the extraction and integration of environment information, whereas dPCC’s computational processes were related to the combination of choice information into DVs for guiding choices. These RSA results, which are orthogonal to those of the univariate analysis in Figures 4, 5, and 6, provide additional support that FPl and dPCC are both involved in environment choice in the form of a functional network.

Finally, we tested more specifically whether FPl involved a parallel processing to extract environment information, which is an important component of the CNN architecture. We systematically varied the number of CNN feature detectors from 1 (i.e., no parallel processing) to 10. Then, we compared the extracted environment information (i.e., the feature maps) of each CNN variant with FPl using an RSA. We found that FPl shared similar representations to all CNN variants with 2–10 feature detectors in parallel (*rho*s > 0.005, signed-rank *P*s < 0.026, permutation *P*s < 0.022; Figure 7C) but not the CNN with only one feature detector (i.e., no parallel processing; *rho* = −0.0002, signed-rank p = 0.977, permutation p = 0.494). In addition, the similarity was greatest to the CNN with four feature detectors, which is the same CNN that was mainly analyzed in this study (*rho* = 0.008, signed-rank p = 0.021, permutation p = 0.012). Taken together, these results suggest FPl adopted a parallel processing for the encoding of environment information.

## Discussion

Environment choice constrains and determines what potential items one will encounter in the future. Choosing a place of residence, an investment plan, or an industry for a career are examples of environment choice that bear long-term consequences for years. However, little is known about the mechanisms of environment choice. Due to its importance for adaptive behavior and its unique computational challenges, we designed a task to measure environment choice and we identified FPl as the neural substrate. To further test the computational mechanisms of FPl during environment choice, we applied a behaviorally optimized and neurally naive CNN. First, we showed that FPl shares similar computations to the CNN, especially in the hidden layers that compute digested information about the environments’ item distributions (Figures 6B and 6C). Second, by systematically comparing a series of CNN variants, we showed that the CNN variant with four parallel feature detectors provides the best account of FPl activity pattern and significantly outperforms an alternative CNN that lacks parallel processing. In other words, extracting multiple environment information simultaneously is an important characteristic of FPl’s computation. Although it remains an empirical question whether FPl contains exactly four separate neuronal populations to integrate different choice features, our results thus far support the view that FPl involves multiple parallel computations to extract complex environment information.

Our findings of FPl, however, offer a different view from the influential neural common currency hypothesis in neuroeconomics. The hypothesis suggests that options of different kinds can be compared biophysically and ubiquitously via the same neural signal.^32^^,^^33^ VmPFC has been a candidate region that signals a neural common currency. Previous fMRI studies suggest that vmPFC activity correlates with the value of a wide range of items, such as money and food.^9^^,^^23^^,^^34^^,^^35^^,^^36^ Recently, this role of vmPFC also received some support from intracranial recording data.^13^ Thus, one might predict the vmPFC signal should be observed in selections both between items and between environments. However, our results revealed a functional dissociation between FPl and vmPFC (Figure 4). As in previous studies, we found that the vmPFC signal emerged during item choice. In contrast, during environment choice, we did not see any vmPFC value signals but a unique signal in FPl. In addition, the neurally naive CNN only shares similar neural representations to the multi-voxel activity pattern of FPl but not to vmPFC. One possibility of the functional dissociation of the vmPFC and FPl is due to the different computational demands of item and environment choices. During item choice, it is critical to compute value based on intrinsic preferences, such that the chosen item would be subjectively rewarding. In contrast, during environment choice, there is a strong computational demand of decomposing complex and abstract choice information and forecasting the long-term consequences of the choices. Hence, we consider this is one reason why the brain engages different regions to fulfill these different computational demands. Taken together, findings suggest that, given the qualitative distinctiveness of environment choice from item choice, the human brain involves an FPl-based mechanism that decomposes and integrates complex information.

Making adaptive environment choice requires prospection or future thinking. People have to envisage the potential items offered in the environment to maximize future rewards. One real-life example is that, while buying houses, people may consider the overall living quality of a certain district before choosing between actual houses within the district. By contrast, whether animals possess the capability of prospection remains as an open question. Despite some prospective behaviors in certain wild animals, such as consuming extra food before hibernation, those behaviors could plausibly arise from inherent predisposition or associative learning.^37^^,^^38^ Experimental evidence also suggests animals lack the reasoning about how their current state is related to the future (e.g., unable to realize tokens as a tool for exchange of rewards in the future^38^). This specificity of prospection in humans should also reasonably be revealed by the presence of a more evolved region in the human brain,^29^^,^^39^ such as Brodmann area 10.

Prospection or future thinking is also involved in other types of decision making, such as model-based choice. For example, if someone wants to buy a specific house, then the person will take a course of earlier actions that lead to the purchase of the house, such as first contacting a specific agency and having a visit to the specific neighborhood. These earlier actions can be seen as proxies to obtain the specific item. Compared with environment choice, model-based choice requires having a model of the transitions between the earlier actions and the specific items, while it also involves less abstract and less complex choice information in these earlier actions. Due to the distinct natures of these related classes of choice, it is important for future studies to contrast neural mechanisms of environment choice and model-based choice.

BA10 that includes FPl is particularly well developed in primates and particularly expanded in human brain.^40^ It has been long described that primates, including humans, have developed a unique granular layer in the prefrontal cortex. A comparison between the human and macaque granular prefrontal cortex showed that FPl in BA10 is uniquely evolved in the human brain.^41^^,^^42^ Intriguingly, most often, BA10 is implicated in cognitive processes related to prospection, such as prospective memory,^7^ mental time travel,^43^ and directed exploration.^44^ Excitation or disruption of BA10 using non-invasive brain stimulation methods also leads to increased and reduced exploration respectively.^44^^,^^45^ All these lines of evidence coincide with our findings about the role of FPl in environment choice.

Despite our current focus on decision between environments, another related line of research that examines stay-switch choices has caught much attention.^46^ This involves studying how people decide between staying with the current items in hand or searching in the environment for better alternatives. Interestingly, these studies generally identify value signals related to the average item or the most rewarding item of the alternative environment in dorsal anterior cingulate cortex (dACC),^27^^,^^29^^,^^47^^,^^48^ a region that is directly connected to BA10.^49^^,^^50^ In a broader sense, dACC and BA10 share a wide range of functional similarities, such as envisioning the choice prospect, hierarchical reasoning, integrating task-relevant information, and monitoring task state.^7^^,^^51^ However, there is little direct contrast in the functional distinction between these two closely connected regions. This study focuses on choices between multiple environments as opposed to many foraging studies that concern average reward rates and environment dynamics, and identified a signal in FPl but not in dACC. It is possible that the FPl specifically concerns future planning of abstract and complex choices, and this may reflect that the evolution of BA10 in primates, especially humans, contributes largely to their behaviors that are particularly abstract and flexible.

Our work revealed that FPl underlies environment choice in contrast to vmPFC, a region that was previously considered as having a universal role in decision making. Crucially, by comparing a behaviorally optimized and neurally naive neural network model and the human neural data, we identified the specific neurocomputations of environment choices; FPl extracts multifaceted environment information via a parallel processing and passes that to dPCC for generating an overall DV. In real life, environment choices, such as buying a house and planning for retirement, often involve pre-selection of options that lead to influential long-term consequences. This ability of decision making between environments to maximize long-term benefits has important survival value. Future studies are warranted to investigate its relationship with the evolution of the human FPl.

### Limitations of the study

Although the task structure of the environment and item choice trials were made very similar, the environment and item options involved stimuli (a series of bars and a single bar, respectively) that were visually different. Since environment choice, by nature, involves more complex information than item choice, it is almost unavoidable that environments need to be represented using more complex visual stimuli. Ideally, the environment and item choices should include identical sets of visual stimuli. One solution was to ask people to first learn the associations between abstract stimuli and the environment/item choice information and then use those abstract stimuli for the subsequent environment/item choice experiment. However, such a design would be cognitively demanding to participants and was avoided in the current study. Another limitation of the current study is that the functional significance of the FPl’s parallel processing is not clear. The feature of parallel processing has been implicated in studies that focused on FPl, such as maintaining multiple goals during multi-tasking.^7^ However, the functional importance of FPl’s parallel processing during environment choice remains elusive. Future experiments that are specifically designed to test FPl’s parallel processing are required to fully address this question.

## STAR★Methods

### Key resources table

**Table:** 

REAGENT or RESOURCE	SOURCE	IDENTIFIER
**Deposited data**		

Behavioral data	This paper	Mendeley Data: https://doi.org/10.17632/6yz4y52sk4.1
fMRI data	This paper	Dyrad: https://doi.org/10.5061/dryad.xsj3tx9m8
Neural Network Data	This paper	Mendeley Data: https://doi.org/10.17632/6yz4y52sk4.1

**Software and algorithms**		

FMRIB’s Software Library	FMRIB, Oxford	https://fsl.fmrib.ox.ac.uk/fsl/fslwiki
MATLAB	MathWorks	https://uk.mathworks.com/
Presentation	Neurobehavioral Systems	https://www.neurobs.com/
VBA toolbox	Daunizeau et al.^52^	https://mbb-team.github.io/VBA-toolbox/

### Resource availability

#### Lead contact

Further information and requests for resources and reagents should be directed to and will be fulfilled by the lead contact, Chun-Kit Law (kelvinck.law@gmail.com).

#### Materials availability

This study did not generate new unique reagents.

### Experimental model and subject details

#### Participants

Twenty-six healthy young adults (15 females), aged 19–39 years with normal or corrected-to-normal vision and no current or history of neurological or psychiatric conditions, were recruited via advertisement in the university and participants’ referral. Written informed consent for each participant was obtained before experiment. This experiment was approved by the Human Subjects Ethics Committee of The Hong Kong Polytechnic University. One participant withdrew because of contraindications to MRI scans. Data of another participant was excluded because the fMRI session had to split into two runs and it was not possible to be analyzed in the same way as the data of other participants. Hence, data of twenty-four participants were analyzed in this study.

### Method details

#### Experimental task

Participants underwent a two-stage decision making task (Figures 1A and 1B). On each Stage 1 trial, participants had to choose between two environments. Each environment was presented in the form of twenty bars and a number (Figure 1B). The height of each bar was related to the reward probability of an item in the environment (i.e. a total of twenty items) and the number indicated the mean reward magnitude of these items. After the selection of an environment, the chosen environment was surrounded by a red frame (0.5 s) and presented at the center (2–7 s). Two items were pseudo-randomly drawn from the chosen environment and offered on the subsequent Stage 2 trial. This was presented visually by highlighting the drawn items in yellow such that the link between the chosen environment and the subsequent items were explicitly illustrated. Then, participants chose between these two items that were associated with different reward magnitudes (between -£10 and £10) and reward probabilities (proportional to the height of the colored bars). The chosen item was then surrounded by a red frame (0.5 s). Reward was delivered according to the magnitude and probability of the chosen item. In this task, participants had to maximize their earning by choosing more rewarding items on Stage 2 trials. In turn, they also had to seek for more advantageous Stage 2 items in advance by choosing a more rewarding environment in Stage 1. To enhance the prolonged influence of Stage 1 choices, participants were told that each chosen Stage 1 environment was randomly followed by up to three Stage 2 trials. Each subsequent Stage 2 trial begun by showing the chosen environment and randomly drawing two items in the environment. After all Stage 2 trials were completed, the rewards associated with the selected items were revealed together. Occasionally, the Stage 2 trials were omitted after an environment was chosen in Stage 1, such that the numbers of Stage 1 trials and Stage 2 trials were comparable in the task. There was a total of 200 Stage 1 trials (400 different environments) and 200 Stage 2 trials (400 different items) in this task. Figures S1B and S1C show the parametric range and autocorrelations of the Stage 1 environments.

Neural signals related to choice values are sometimes confounded by choice salience because they are collinear when only rewarding options are included in an experiment.^4^^,^^20^ To avoid this, this task included environments and items that were related to gains (Figure 1C; green bars) and losses (red bars), which is an effective way of orthogonalising value and salience. 53.5% (55%) of the trials involved two gain environments (items), 31.5% (30%) of the trials involved two loss environments (items) and 15% of the trials involved one gain and one loss environment (item).

Human choice is flexible to contextual demands and neural signal that reflects choice values should also be flexibly modulated in the same manner. To examine the flexibility of neural signals related to environment and item choices, there were two Bonus Conditions in this task (Figure 1C). In each block, starting from Stage 1 a bonus (between -£4 and £6) was displayed at the lower center. The bonus value and Bonus Condition stayed the same on the subsequent Stage 2 trials of the same block. In Linked Condition (indicated by two arrows pointing from the bonus to the two environments/items), the bonus was obtained when the chosen Stage 2 item led to reward. Hence, in the presence of bonuses that were particularly rewarding, participants were incentivized to choose environments composed of items with larger probabilities, such that eventually they would be offered these items to win the bonus. In contrast, in Unlinked Condition (indicated by crosses), the bonus was delivered unconditionally (i.e. independent of the probabilistic choice outcomes) such that participants’ choices ought not to be affected by the bonus.

#### Behavioral training session

Before fMRI scanning, all participants underwent a training session to get familiar with the experimental task. First, the experimenter explained the task instruction in details, including how the reward magnitude and probability of environments/items were represented, the relationship between Stage 1 and Stage 2, the differences between conditions (Gain vs. Loss Condition, Linked vs. Unlinked Bonus Condition), and the way of obtaining the bonus. Participants were explicitly told that the final incentive depended on the choice outcomes of all trials in the experiment. Afterward, participants underwent a practice task which was a shortened version of the actual experimental task (51 Stage 1 trials, 60 Stage 2 trials). The choice accuracy in Stage 1 trials was significantly greater in the actual experimental session than that in the training session (*t*_23_ = 5.971, p = 4.355 × 10^−6^), while that in Stage 2 trials remained similar (*t*_23_ = 0.141, p = 0.889).

### Quantification and statistical analysis

#### Behavioral analysis

To test whether participants’ choices (to the right = 1 and left = 0) were biased by the options’ expected value and probability, bonus value, and Bonus Condition, General Linear Model (GLM) 1 (GLM1) was applied to analyze the data of Stage 1 environment choice and Stage 2 item choice separately:

$$logit\frac{P\left(R\right)}{1-P\left(R\right)}=\beta_{0}+\beta_{1}{EV}_{\left(R-L\right)}+\beta_{2}Prob_{\left(R-L\right)}+\beta_{3}Bon+\beta_{4}Cond+\beta_{5}\left({EV}_{\left(R-L\right)}\times Bon\times Cond\right)+\beta_{6}\left(Prob_{\left(R-L\right)}\times Bon\times Cond\right)$$

where *P(R)* denotes the probability of choosing the rightward option. *Prob*_*(R-L)*_ denotes the difference in reward probabilities (i.e. the average environment probability in Stage 1 or item probability in Stage 2) between the rightward and leftward options; *EV*_*(R-L)*_ denotes the difference in expected value (reward magnitude multiplied by reward probability) between the rightward and leftward options; *Bon* and *Cond* represent the bonus value and Bonus Condition (Linked Condition = 1, Unlinked Condition = 0) respectively.

Each Stage 1 environment consisted of a distribution of twenty items. To test whether participants’ environment choices were biased by the statistical moments of the item distributions, GLM2 was performed:

$$logit\frac{P\left(R\right)}{1-P\left(R\right)}=\beta_{0}+\beta_{1}{EV}_{\left(R-L\right)}+\beta_{2}{Mean}_{\left(R-L\right)}+\beta_{3}{Variance}_{\left(R-L\right)}+\beta_{4}{Skewness}_{\left(R-L\right)}$$

where *Mean*_*(R-L)*_, *Variance*_*(R-L)*_, *Skewness*_*(R-L)*_ denote the differences in mean, variance, and skewness of environment probability respectively. A one-sample t test was performed to examine whether each beta weight was significantly different from zero across participants in both GLMs.

#### Computational modeling

##### Stage 1 CNN for environment choice

Environment choice requires integration of complex information about item distribution. One characteristic of the convolutional neural network (CNN) is its capability to integrate bundles of information into digested feature representation. Therefore, we employed the CNN to investigate how complex environment values were evaluated and integrated to give rise to decisions. There are four essential layers in the CNN: (1) input layer; (2) hidden layers; (3) choice-predicting fully-connected layer; (4) output layer (Figures 3A and S4Ai). First, in the input layer, environment values (i.e. reward magnitude and reward probability), and bonus information are fed in as the input of the CNN. Reward probability in the input layer (represented in a matrix) is convolved by four feature detectors and transformed into four sets of feature maps. During convolution, each feature detector that contains a set of weights, slides along the input matrix of reward probability of the environments. In each stride, the superimposed portion in the input is multiplied by the feature detector weights and the weighted-sum becomes the output of that stride. A feature map generated from this convolution process can be formulated as follow:

$${Feature\;map}_{ij}^{f}=b_{f}^{\left(1\right)}+\sum_{m=1}^{M}\sum_{n=1}^{N}W_{mn}^{\left(1\right)}⊙X_{\left(i+m-1\right)\left(j+n-1\right)}$$

where *X* is the reward probability matrix with a size of *I*×*J*; *W* is the matrix of the feature detector weights with a size of *M*×*N* and *W*^*(1)*^_*mn*_ refers to the element in the *m*^*th*^ row, *n*^*th*^ column; *b*^*(1)*^_*f*_ is the bias term for the *f*^*th*^ feature map; The row and column of the feature map are denoted by *i* and *j* respectively (*i*∈{1,2, …,[*I*-*m*+1]}; *j*∈{1,2, …,[*J*-*n*+1]}).

After convolution, the feature maps related to the reward probabilities, the reward magnitudes, the product between the feature maps and reward magnitudes, and bonus information are concatenated (indicated as *concat*; Figure S4Ai). Next, *concat* is vectorized in the first fully-connected layer (*fc1*) and transformed into 512 nodes and then activated by a rectified linear unit (ReLU) function:

$$Y_{N}=W_{N}^{\left(2\right)}．concat+b_{N}^{\left(2\right)}$$

$$ReLU\left(Y\right)=\left\{\begin{array}{c} 0\;if\;Y<0\\ Y\;if\;Y\geq 0 \end{array}\right\}$$

where *W*^*(2)*^_*N*_ and *b*^*(2)*^_*N*_ are the weight matrix and bias term for the *N*^*th*^ node respectively. In the subsequent choice-predicting fully-connected layer (*final fc*), all nodes are concatenated into one column vector and then weigh-summed to form the decision values (DVs). The DVs for the *k*^*th*^ environment are computed as follow:

$$DV_{k}=W_{k}^{\left(3\right)}\cdot Y+b_{k}^{\left(3\right)}$$

where *W*^*(3)*^_*k*_ and *b*^*(3)*^_*k*_ are the weight matrix and bias term for the *k*^*th*^ environment respectively. Finally, the choice probability of each environment is computed by applying a softmax function to the DVs in the output layer:

$$P\left(Environment_{k}\right)=\frac{e^{{DV}_{k}}}{\sum_{k}^{2}e^{DV_{k}}}$$

##### “Simplified” CNNs

To investigate whether the Stage 1 CNN integrates information about statistical moments of the environments (Figure 7C), we developed five “simplified” CNN models, namely Mean Model, Mean+Var Model, Mean+Skew Model, Var+Skew Model, and Mean+Var+Skew Model (Figure 7C). In the original Stage 1 CNN, the values of all component items in each environment are individually specified in the input. In contrast, in the Mean Model, the input involves the mean of the items’ value, instead of individual item values. In the Mean+Var Model, the input involves the mean and variance of item value. In the Mean+Skew Model, the input involves the mean and skewness of item value. In the Var+Skew Model, the input involves the variance and skewness of item value. In the Mean+Var+Skew Model, the input involves the mean, variance and skewness of item value.

##### Partial DV contributed by each feature map

In the CNN model, the DVs in the final fully-connected layer are weighted combinations of the information extracted by four feature detectors. To test the specific information represented by each feature detector, we calculated the “partial DV” of each feature detector. This was achieved by simply keeping the information from one feature detector at a time, while discarding the information from all remaining feature detectors.

##### Stage 2 ANN for item choice

An artificial neural network (ANN) was applied to model Stage 2 item choice behavior (Figure S4Bi). It is largely similar to the Stage 1 CNN except that it involves item values as the input and an absence of convolution.

##### General linear model (GLM)

In addition to the CNN, we performed five additional models to identify the model that provides the best account of participants’ choice behavior. The first one is the GLM which was adapted from GLM1, the DV was fitted using the following equation:

$$DV_{R}=\beta_{0}+\beta_{1}Mag_{\left(R-L\right)}+\beta_{2}Prob_{\left(R-L\right)}+\beta_{3}Bon+\beta_{4}Cond+\beta_{5}\left(Mag_{R}\times Prob_{R}-Mag_{L}\times Prob_{L}\right)+\beta_{6}\left(Prob_{\left(R-L\right)}\times Bon\times Cond\right)$$

where *DV*_*R*_ is the DV of the rightward option (environment in Stage 1 or item in Stage 2); *Mag*_*(R-L)*_ denotes the difference in reward magnitude between the rightward and leftward options. All other variables are identical to those included in GLM1: *Prob*_*(R-L)*_ denotes the difference in reward probabilities between the rightward and leftward options; *Bon* and *Cond* represent the bonus value and Bonus Condition (Linked Condition = 1, Unlinked Condition = 0) respectively.

##### Mean-variance-skewness (MVS) model

The MVS model^53^^,^^54^^,^^55^^,^^56^ was applied to estimate the subjective probability of a given environment based on the mean, variance, and skewness of its component items:

$$\hat{Prob^{k}}=\frac{1}{20}\sum_{q=1}^{20}{Prob}_{q}^{k}+\rho Var\left({Prob}^{k}\right)+\sigma Skew\left({Prob}^{k}\right)$$

where $\hat{Prob^{k}}$ denotes the subjective probability of the *k*^th^ environment (either the environment on the left or right); *Prob*^*k*^_*q*_ denotes the *q*^th^ individual item probability in the *k*^th^ environment; *ρ* and *σ* are free parameters that denote the preferences for variance and skewness respectively. DV of the *k*^th^ environment is the product of its reward magnitude (*Mag*^*k*^) and subjective probability:

$${DV}_{k}=\beta_{1}Mag^{k}+\hat{\beta_{2}Prob^{k}}+\beta_{3}Bon+\beta_{4}Cond+\beta_{5}(Mag^{k}\times Prob^{k}+\beta_{6}\left(\hat{Prob^{k}}\times Bon\times Cond\right)$$

##### Power law model

Power law was shown applicable to the estimation of the average value of a distribution.^57^ Hence, we adapted the power law to estimate the subjective probabilities of environments as follow:

$$\hat{Prob^{k}}=\frac{1}{20}\sum_{q=1}^{20}{{(Prob}_{q}^{k})}^{g}$$

where each individual item probability in an environment is transduced by a power *g*. *DV* of the *k*^*th*^ environment is identical to that of MVS model.

##### Cumulative prospect theory

We also adopted a classical model of subjective value estimation towards economic values, the cumulative prospect theory,^58^ to estimate non-linearly the subjective probabilities and magnitudes of environments.

$$\hat{Mag^{k}}=\left\{\begin{array}{c} {\left({Mag}^{k}\right)}^{\alpha }\;if\;{Mag}^{k}\geq 0\\ {-\lambda \left(-{Mag}^{k}\right)}^{\beta }\;if\;{Mag}^{k}<0 \end{array}\right\}$$

where *Mag*^*k*^ is the reward magnitude of the *k*^*th*^ option and $\hat{Mag^{k}}$ is the resultant subjective reward magnitude. Sensitivity to value change under gains and losses is denoted by *α* and *β* respectively whereas the degree of loss aversion is indicated by *λ*. Besides, subjective probability under gains and losses is estimated via two weighting functions:

$$\hat{Prob^{k}}=\left\{\begin{array}{c} \frac{1}{20}\sum_{q=1}^{20}\frac{{\left({Prob}_{q}^{k}\right)}^{\gamma }}{({\left({Prob}_{q}^{k}\right)}^{\gamma }+{{\left(1-{Prob}_{q}^{k}\right)}^{\gamma })}^{\frac{1}{\gamma }}}\;if\;{Mag}^{k}\geq 0\\ \frac{1}{20}\sum_{q=1}^{20}\frac{{\left({Prob}_{q}^{k}\right)}^{\Delta }}{({\left({Prob}_{q}^{k}\right)}^{\Delta }+{{\left(1-{Prob}_{q}^{k}\right)}^{\Delta })}^{\frac{1}{\Delta }}}\;if\;{Mag}^{k}<0 \end{array}\right\}$$

where the degrees of upweighting of small probability and downweighting of large probability under gains and losses are indicated by *γ* and *Δ* respectively. Computation of *DV* of the *k*^*th*^ option is similar to Equation 3 except subjective reward magnitude ($\hat{Mag^{k}}$) is used:

$$DV_{R}=\beta_{1}\hat{{Mag}^{k}}+\beta_{2}\hat{Prob^{k}}+\beta_{3}Bon+\beta_{4}Cond+\beta_{5}\hat{({Mag}^{k}}\times \hat{Prob^{k}})+\beta_{6}\left(\hat{Prob^{k}}\times Bon\times Cond\right)$$

##### Autoencoder

Apart from the CNN, we applied another deep learning neural network, the autoencoder, to fit participants’ choices. Similar to the CNN, option value in terms of reward magnitude (Mag), reward probability (Prob), bonus value (Bon), Bonus Condition (Cond), expected value (Mag×Prob), and Prob×Bon×Cond are fed in as the input of the autoencoder. The autoencoder transforms the input into 512 hidden nodes and each hidden node is activated by a sigmoid function:

$$Z_{h}=Sigmoid\left(W_{h}^{\left(4\right)}\cdot X_{Autoenc}+b^{\left(4\right)}\right)$$

$$Sigmoid\left(z\right)=\frac{1}{1+e^{-z}}$$

where *Z*_*h*_ is the *h*^*th*^ hidden node; *X*_*Autoenc*_ is the input; *W*^*(4)*^_*h*_ is the weight matrix for the *h*^*th*^ node; *b*^*(4)*^ is a bias term. The *DV* for the *k*^*th*^ option is computed by:

$$DV_{k}=W_{k}^{\left(5\right)}\cdot Z+b_{k}^{\left(5\right)}$$

where *W*^*(5)*^_*k*_ and *b*_*k*_^*(5)*^ are the weight matrix and the bias term for the *k*^th^ option respectively.

Note that we tested different autoencoder architectures by systematically varying the number of hidden nodes (i.e.1-42, 64, 128, 256, 512), as in what was performed for the CNN (Figure S4), and the prediction accuracies ranged from 51% to 87%. The variant with 512 hidden nodes yielded the lowest negative log likelihood (NLL; 37.175) and thus was selected for comparison with other computational models.

##### Parameter optimization and model comparison

All CNNs (Stage 1 and Simplified) and ANN were fitted to participants’ choices using MATLAB Deep Learning Toolbox (The MathWorks, 2021) whereas the alternative models were fitted using MATLAB VBA-toolbox.^52^ We applied a 20-fold cross-validation procedure for each model to prevent overfitting. Specifically, we collapsed all the choices made from all participants and then randomly split them into 20 subsets. All subsets except one were used for training the model and the remaining subset was used for testing the trained model. This procedure repeated 20 times and the testing subset was changed every time. The goodness-of-fit of models were compared by their negative log likelihood (NLL) via independent samples t-tests.

#### Neuroimaging data acquisition and preprocessing

Neuroimaging data were collected by a Siemens 3T MRI scanner. FMRI data was collected with a multi-band echo planar imaging sequence: 2.5 × 2.5 × 2.5 mm^3^ voxel-resolution, TR = 1.6s, TE1 = 15ms, TE2 = 36.19ms, TE3 = 57.38ms, flip angle = 70°, slice angle = −30° (T>C). Field maps were collected to correct for signal distortions using a dual echo 2D gradient echo sequence: 2.5 × 2.5 × 2.5 mm^3^ voxel resolution, TR = 590ms, TE1 = 4.92ms, TE2 = 7.38ms. T1-weighted structural images were acquired using an MPRAGE sequence: 1 × 1 × 1 mm^3^ voxel resolution, 174 × 192 × 192 grid, TR = 1900ms, TE = 3.97ms, TI = 904ms.

FMRI data was analyzed using FMRIB’s Software Library (FSL).^59^ The following procedures were used to preprocessed the fMRI data: brain extraction by Brain Extraction Tool,^60^ motion correction by using FMRIB’s Linear Image Registration Tool,^61^ Gaussian spatial smoothing with fullwidth at half maximum of 5mm, field map correction for distorted signal,^62^ and high-pass temporal filtering (3 dB cutoff of 100s). FMRI data were registered to each participant’s high-resolution structural image and then normalized into the standard Montreal Neurological Institute (MNI) space).^63^

#### Whole-brain analysis

All whole-brain analyses were performed using a univariate GLM approach. To investigate the value signals during environment choice and item choice, GLM3 was applied to analyze the fMRI data of individual participants, which involves the following two regressors: ΔDV_Env_ (i.e. difference in DV between the chosen and unchosen Stage 1 environments) and ΔDV_Item_ (i.e. difference in DV between the chosen and unchosen Stage 2 items). These terms were time-locked to the onset time when the environments/items were offered and convolved with a haemodynamic response function. Six additional nuisance regressors were also included. One parametric regressor related to the bonus value, time-locked to block bonus onset, was included. One parametric regressor related to the value of the chosen environment, time-locked to the onset of Stage 1 delay phase, was included to model the neural signal related to the chosen environment prior to the onset of each Stage 2 trial. Two box car regressors related to left-hand and right-hand responses, time-locked to participants’ decisions, were included. Finally, two regressors related to the average BOLD signal in the cerebrospinal fluid (CSF) and white matter (WM) were included. At group level, FMRIB’s local analysis of mixed effects^64^^,^^65^ was applied with outlier deweighting.^66^ A cluster-based thresholding of Z > 3.1 and a significance threshold of p < 0.05 were used for identifying significant clusters,^67^ unless otherwise specified.

#### Region of interest (ROI) analysis

ROI analysis was performed to scrutinize the time courses of the key brain regions identified from the whole-brain analysis. To avoid circularity, all ROIs were located based on the findings of previous studies that revealed similar cognitive processes. From GLM3, we found that signals in the lateral frontopolar cortex (FPl) and the ventromedial prefrontal cortex (vmPFC) were related to ΔDV during environment choice and item choice respectively. A mask was created to extract the activity of the FPl by centering a sphere of 3mm radius at the coordinates that is implicated in directed exploration,^44^ whereas activity of the vmPFC was extracted by centering a sphere of 3mm radius at the coordinates that is related to item choice.^68^ GLM3 also identified the dorsal posterior cingulate cortex (dPCC) during both environment and item choices. A mask that was developed based on reward-guided decision making^69^ was used to extract the activity of the dPCC. The extracted ROI time courses were then upsampled ten-times by cubic spline interpolation and time-locked to stimulus onset in Stage 1 environment choice and Stage 2 item choice respectively. For each participant, the ROI activities at each time point were regressed via a GLM, such that time courses of beta weight for each regressor in the GLM were acquired. The beta weight time courses were then group averaged and plotted (e.g. Figure 4B). The size of each peak was extracted from every participant, and then a one-sample t test was performed to test whether each peak in the group time courses significantly differed from zero. Since traditional frequentists statistics is not ideal for confirming null effects, each analysis was also supplemented by a Bayesian t test. A leave-one-subject-out procedure was applied to avoid any bias during the estimation of the peak size.^70^^,^^71^ Specifically, the full-width half-maximum of a peak in the group time course defined the time window for extracting that peak. Moreover, for a given participant, the time point of peak extraction was defined by the peak’s position within this time window when the group time course excluded the beta weights from that participant. The peak for statistical tests was extracted by repeating this leave-one-out procedure for all participants.

In Figure 6A, bonus adaptation (i.e. differential responses in different Bonus Conditions) was tested by GLM4. It included three regressors: (1) a DV term estimated using all choice information except the bonus (DV_Basic_); (2) a DV term estimated using only the bonus value in the Linked Condition (DV_Linked_ _Bonus_) and; (3) a DV term estimated using only the bonus value in the Unlinked Condition, but the bonus was included as if it was in the Linked Condition (DV_Unlinked Bonus_). For vmPFC in Stage 2 item choice (Figure 6Aii), GLM5 was performed by including two additional regressors into GLM4: a binary term that describes whether the trial was the first item trial of the block (first trial = 1, non-first trial = −1) and its interaction term with the ΔDV_Linked Bonus_. In Figure 6B, salience invariance was tested using GLM6. Regressors included ΔDV, salience (i.e. the sum of the two options’ absolute values), and DV sum (a nuisance regressor to account for the remaining variance associated with the DV of the options). Note that in GLM6, salience and DV sum were calculated by EV instead of the CNN DV. This was because the signs of the option values were lost in the CNN DV during the normalization and non-linear transformation procedures.

#### Psychophysiological interaction (PPI) analysis

PPI analysis was carried out to test the functional coupling of the dPCC with FPl and vmPFC (Figure 5). The dPCC was defined as the seed region and the PPI included the following regressors: (1) a psychological regressor about the stage of the task (a dummy variable; 1 = Stage 1 environment choice, 0 = Stage 2 item choice); (2) a physiological regressor of the FPl time courses; (3) a physiological regressor of the vmPFC time courses; (4) an interaction term between regressors (1) and (2); (5) an interaction term between regressors (1) and (3).

#### Representational similarity analysis (RSA)

RSA^72^ was performed to test the similarity in computational representations between different datasets, including fMRI neural data and CNN predictions, by comparing their representational dissimilarity matrices (RDMs). We first computed the RDMs of the neural data or CNN predictions for each participant. Thereafter, RSAs were conducted in a participant-by-participant manner.

For CNN models, the trial-by-trial nodal activations of all layers or a selected layer (e.g. input layer) were extracted. Next, a self-correlation was performed using Pearson correlation and then subtracted from 1 to become an RDM of that layer. On the other hand, for fMRI neural data, RDM computation follows the same procedures except that trial-by-trial voxel activations in different ROIs were extracted instead. In addition to the FPl, voxel activation of other ROIs and control regions, namely the vmPFC, dPCC, primary visual cortex (V1), and CSF, were also extracted. Voxel activation extraction for V1 and CSF was carried out using atlases available in FSL.^59^ The masks for FPl, vmPFC, and dPCC are described in the previous section ROI analysis. Next, to examine the computational similarity between different neural data and CNN predictions, RSAs were performed by computing Spearman correlations between different pairs of RDMs. Note that the upper and lower triangles within an RDM (separated by the diagonal) are always symmetrical. Hence, only the upper triangle was included for RSA. All RSA correlation coefficients were compared via signed-rank tests and permutation tests.

It is worth noting that one participant did not fully complete the decision making task within an fMRI run such that there were fewer number of trials for neural data than behavioral data. When RSA between neural RDM and CNN RDM was performed for that participant, the CNN RDM was computed excluding the trials that lacked fMRI data.

#### Additional behavioral experiment

##### Participants

Twenty healthy young adults (11 females), aged 18–27 years with normal or corrected-to-normal vision and no current or history of neurological or psychiatric conditions, were recruited via advertisement in the university and participants’ referral. Written informed consent of each participant was obtained before experiment. The experiment was approved by the Human Subjects Ethics Committee of the Hong Kong Polytechnic University.

##### Experimental task

It was a simplified environment choice task, which is similar to the Stage 1 of the main experiment (Figure S2A). In each block, participants chose repeatedly between two environments for ten trials. The environments were similar to those in the main experiment – each of which was presented in a form of a distribution of twenty bars (representing reward probabilities of twenty items) and a number (the mean reward magnitude of the items; from -HK$100 to HK$100). Once a decision was made, the chosen environment was surrounded by a blue frame (0.5 s). After a delay of 0.7 s, an item from the chosen environment was randomly drawn and highlighted in yellow. Probabilistic reward was delivered according to the drawn item. When all ten trials of the same block were completed, decision outcomes were shown at once. There were 400 trials in total.

## Data Availability

• All reported data have been deposited at Mendeley and Dryad and are publicly available as of the date of publication. DOIs are listed in the key resources table. • All original code has been deposited at Mendeley and is publicly available as of the date of publication. DOIs are listed in the key resources table. • Any additional information required to reanalyze the data reported in this paper is available from the lead contact upon request.

## Supplemental information

Document S1. Figures S1–S6 and Tables S1 and S2

Document S2. Article plus supplemental information

## References

[1] Chau B.K., Law C.-K., Lopez-Persem A., Klein-Flügge M.C., Rushworth M.F. (2020). Consistent patterns of distractor effects during decision making. Elife.

[2] Walton M.E., Chau B.K., Kennerley S.W. (2015). Prioritising the relevant information for learning and decision making within orbital and ventromedial prefrontal cortex. Curr. Opin. Behav. Sci..

[3] Woo T.-F., Law C.-K., Ting K.-H., Chan C.C.H., Kolling N., Watanabe K., Chau B.K.H. (2021). Distinct causal influences of dorsolateral prefrontal cortex and posterior parietal cortex in multiple-option decision making. Cerebr. Cortex.

[4] Bartra O., McGuire J.T., Kable J.W. (2013). The valuation system: a coordinate-based meta-analysis of BOLD fMRI experiments examining neural correlates of subjective value. Neuroimage.

[5] Kim C., Kroger J.K., Calhoun V.D., Clark V.P. (2015). The role of the frontopolar cortex in manipulation of integrated information in working memory. Neurosci. Lett..

[6] Koechlin E., Hyafil A. (2007). Anterior prefrontal function and the limits of human decision-making. Science.

[7] Mansouri F.A., Koechlin E., Rosa M.G.P., Buckley M.J. (2017). Managing competing goals — a key role for the frontopolar cortex. Nat. Rev. Neurosci..

[8] Abitbol R., Lebreton M., Hollard G., Richmond B.J., Bouret S., Pessiglione M. (2015). Neural mechanisms underlying contextual dependency of subjective values: converging evidence from monkeys and humans. J. Neurosci..

[9] Castegnetti G., Zurita M., De Martino B. (2021). How usefulness shapes neural representations during goal-directed behavior. Sci. Adv..

[10] Chau B.K.H., Kolling N., Hunt L.T., Walton M.E., Rushworth M.F.S. (2014). A neural mechanism underlying failure of optimal choice with multiple alternatives. Nat. Neurosci..

[11] Fouragnan E.F., Chau B.K.H., Folloni D., Kolling N., Verhagen L., Klein-Flügge M., Tankelevitch L., Papageorgiou G.K., Aubry J.-F., Sallet J. (2019). The macaque anterior cingulate cortex translates counterfactual choice value into actual behavioral change. Nat. Neurosci..

[12] Lebreton M., Jorge S., Michel V., Thirion B., Pessiglione M. (2009). An automatic valuation system in the human brain: evidence from functional neuroimaging. Neuron.

[13] Lopez-Persem A., Bastin J., Petton M., Abitbol R., Lehongre K., Adam C., Navarro V., Rheims S., Kahane P., Domenech P. (2020). Four core properties of the human brain valuation system demonstrated in intracranial signals. Nat. Neurosci..

[14] McNamee D., Rangel A., O’Doherty J.P. (2013). Category-dependent and category-independent goal-value codes in human ventromedial prefrontal cortex. Nat. Neurosci..

[15] Eslinger P.J., Damasio A.R. (1985). Severe disturbance of higher cognition after bilateral frontal lobe ablation: patient EVR. Neurology.

[16] Shallice T., Burgess P.W. (1991). Deficits in strategy application following frontal lobe damage in man. Brain.

[17] Levy D.J., Glimcher P.W. (2012). The root of all value: a neural common currency for choice. Curr. Opin. Neurobiol..

[18] Juechems K., Balaguer J., Herce Castañón S., Ruz M., O’Reilly J.X., Summerfield C. (2019). A network for computing value equilibrium in the human medial prefrontal cortex. Neuron.

[19] Kolling N., Behrens T., Wittmann M., Rushworth M. (2016). Multiple signals in anterior cingulate cortex. Curr. Opin. Neurobiol..

[20] Litt A., Plassmann H., Shiv B., Rangel A. (2011). Dissociating valuation and saliency signals during decision-making. Cerebr. Cortex.

[21] Krizhevsky A., Sutskever I., Hinton G.E. (2017). ImageNet classification with deep convolutional neural networks. Commun. ACM.

[22] Lindsay G.W. (2021). Convolutional neural networks as a model of the visual system: past, present, and future. J. Cogn. Neurosci..

[23] Levy D.J., Glimcher P.W. (2011). Comparing apples and oranges: using reward-specific and reward-general subjective value representation in the brain. J. Neurosci..

[24] Boorman E.D., Behrens T.E.J., Woolrich M.W., Rushworth M.F.S. (2009). How green is the grass on the other side? Frontopolar cortex and the evidence in favor of alternative courses of action. Neuron.

[25] Boorman E.D., Rushworth M.F., Behrens T.E. (2013). Ventromedial prefrontal and anterior cingulate cortex adopt choice and default reference frames during sequential multi-alternative choice. J. Neurosci..

[26] Hunt L.T., Kolling N., Soltani A., Woolrich M.W., Rushworth M.F.S., Behrens T.E.J. (2012). Mechanisms underlying cortical activity during value-guided choice. Nat. Neurosci..

[27] Kolling N., Behrens T.E.J., Mars R.B., Rushworth M.F.S. (2012). Neural mechanisms of foraging. Science.

[28] Lopez-Persem A., Domenech P., Pessiglione M. (2016). How prior preferences determine decision-making frames and biases in the human brain. Elife.

[29] Kolling N., Scholl J., Chekroud A., Trier H.A., Rushworth M.F.S. (2018). Prospection, perseverance, and insight in sequential behavior. Neuron.

[30] Suzuki S., Adachi R., Dunne S., Bossaerts P., O’Doherty J.P. (2015). Neural mechanisms underlying human consensus decision-making. Neuron.

[31] Haynes J.-D., Sakai K., Rees G., Gilbert S., Frith C., Passingham R.E. (2007). Reading hidden intentions in the human brain. Curr. Biol..

[32] Padoa-Schioppa C. (2011). Neurobiology of economic choice: a good-based model. Annu. Rev. Neurosci..

[33] Zhang Z., Fanning J., Ehrlich D.B., Chen W., Lee D., Levy I. (2017). Distributed neural representation of saliency controlled value and category during anticipation of rewards and punishments. Nat. Commun..

[34] Chib V.S., Rangel A., Shimojo S., O’Doherty J.P. (2009). Evidence for a common representation of decision values for dissimilar goods in human ventromedial prefrontal cortex. J. Neurosci..

[35] FitzGerald T.H.B., Seymour B., Dolan R.J. (2009). The role of human orbitofrontal cortex in value comparison for incommensurable objects. J. Neurosci..

[36] Kim H., Shimojo S., O’Doherty J.P. (2011). Overlapping responses for the expectation of juice and money rewards in human ventromedial prefrontal cortex. Cerebr. Cortex.

[37] Baumeister R.F., Lim K., Glăveanu V.P. (2021). The Palgrave Encyclopedia of the Possible.

[38] Redshaw J., Bulley A., Oettingen G., Sevincer A.T., Gollwitzer P. (2018). The Psychology of Thinking about the Future.

[39] Scholl J., Kolling N., Nelissen N., Wittmann M.K., Harmer C.J., Rushworth M.F.S. (2015). The good, the bad, and the irrelevant: neural mechanisms of learning real and hypothetical rewards and effort. J. Neurosci..

[40] Semendeferi K., Armstrong E., Schleicher A., Zilles K., Van Hoesen G.W. (2001). Prefrontal cortex in humans and apes: a comparative study of area 10. Am. J. Phys. Anthropol..

[41] Koechlin E. (2011). Frontal pole function: what is specifically human?. Trends Cogn. Sci..

[42] Neubert F.-X., Mars R.B., Thomas A.G., Sallet J., Rushworth M.F.S. (2014). Comparison of human ventral frontal cortex areas for cognitive control and language with areas in monkey frontal cortex. Neuron.

[43] Suddendorf T., Corballis M.C. (2007). The evolution of foresight: what is mental time travel, and is it unique to humans? Behav. Brain Sci..

[44] Zajkowski W.K., Kossut M., Wilson R.C. (2017). A causal role for right frontopolar cortex in directed, but not random, exploration. Elife.

[45] Raja Beharelle A., Polania R., Hare T.A., Ruff C.C. (2015). Transcranial stimulation over frontopolar cortex elucidates the choice attributes and neural mechanisms used to resolve exploration-exploitation trade-offs. J. Neurosci..

[46] Mobbs D., Hassabis D., Yu R., Chu C., Rushworth M., Boorman E., Dalgleish T. (2013). Foraging under competition: the neural basis of input-matching in humans. J. Neurosci..

[47] Hayden B.Y., Pearson J.M., Platt M.L. (2011). Neuronal basis of sequential foraging decisions in a patchy environment. Nat. Neurosci..

[48] Sarafyazd M., Jazayeri M. (2019). Hierarchical reasoning by neural circuits in the frontal cortex. Science.

[49] Carmichael S.T., Price J.L. (1996). Connectional networks within the orbital and medial prefrontal cortex of macaque monkeys. J. Comp. Neurol..

[50] Saleem K.S., Miller B., Price J.L. (2014). Subdivisions and connectional networks of the lateral prefrontal cortex in the macaque monkey: connections of the lateral prefrontal cortex. J. Comp. Neurol..

[51] Heilbronner S.R., Hayden B.Y. (2016). Dorsal anterior cingulate cortex: a bottom-up view. Annu. Rev. Neurosci..

[52] Daunizeau J., Adam V., Rigoux L. (2014). VBA: a probabilistic treatment of nonlinear models for neurobiological and behavioural data. PLoS Comput. Biol..

[53] Symmonds M., Wright N.D., Bach D.R., Dolan R.J. (2011). Deconstructing risk: separable encoding of variance and skewness in the brain. Neuroimage.

[54] Wright N.D., Symmonds M., Hodgson K., Fitzgerald T.H.B., Crawford B., Dolan R.J. (2012). Approach-avoidance processes contribute to dissociable impacts of risk and loss on choice. J. Neurosci..

[55] Wright N.D., Symmonds M., Morris L.S., Dolan R.J. (2013). Dissociable influences of skewness and valence on economic choice and neural activity. PLoS One.

[56] Wright N.D., Symmonds M., Dolan R.J. (2013). Distinct encoding of risk and value in economic choice between multiple risky options. Neuroimage.

[57] Li V., Herce Castañón S., Solomon J.A., Vandormael H., Summerfield C. (2017). Robust averaging protects decisions from noise in neural computations. PLoS Comput. Biol..

[58] Tversky A., Kahneman D. (1992). Advances in prospect theory: cumulative representation of uncertainty. J. Risk Uncertain..

[59] Smith S.M., Jenkinson M., Woolrich M.W., Beckmann C.F., Behrens T.E.J., Johansen-Berg H., Bannister P.R., De Luca M., Drobnjak I., Flitney D.E. (2004). Advances in functional and structural MR image analysis and implementation as FSL. Neuroimage.

[60] Smith S.M. (2002). Fast robust automated brain extraction. Hum. Brain Mapp..

[61] Jenkinson M., Bannister P., Brady M., Smith S. (2002). Improved optimization for the robust and accurate linear registration and motion correction of brain images. Neuroimage.

[62] Jenkinson M. (2003). Fast, automated,N-dimensional phase-unwrapping algorithm. Magn. Reson. Med..

[63] Jenkinson M., Smith S. (2001). A global optimisation method for robust affine registration of brain images. Med. Image Anal..

[64] Beckmann C.F., Jenkinson M., Smith S.M. (2003). General multilevel linear modeling for group analysis in FMRI. Neuroimage.

[65] Woolrich M.W., Behrens T.E.J., Beckmann C.F., Jenkinson M., Smith S.M. (2004). Multilevel linear modelling for FMRI group analysis using Bayesian inference. Neuroimage.

[66] Woolrich M. (2008). Robust group analysis using outlier inference. Neuroimage.

[67] Worsley K.J., Evans A.C., Marrett S., Neelin P. (1992). A three-dimensional statistical analysis for CBF activation studies in human brain. J. Cerebr. Blood Flow Metabol..

[68] Juechems K., Balaguer J., Ruz M., Summerfield C. (2017). Ventromedial prefrontal cortex encodes a latent estimate of cumulative reward. Neuron.

[69] Neubert F.-X., Mars R.B., Sallet J., Rushworth M.F.S. (2015). Connectivity reveals relationship of brain areas for reward-guided learning and decision making in human and monkey frontal cortex. Proc. Natl. Acad. Sci. USA.

[70] Chau B.K.H., Sallet J., Papageorgiou G.K., Noonan M.P., Bell A.H., Walton M.E., Rushworth M.F.S. (2015). Contrasting roles for orbitofrontal cortex and amygdala in credit assignment and learning in macaques. Neuron.

[71] Trudel N., Scholl J., Klein-Flügge M.C., Fouragnan E., Tankelevitch L., Wittmann M.K., Rushworth M.F.S. (2021). Polarity of uncertainty representation during exploration and exploitation in ventromedial prefrontal cortex. Nat. Human Behav..

[72] Kriegeskorte N. (2008). Representational similarity analysis – connecting the branches of systems neuroscience. Front. Syst. Neurosci..

