# Supplementary material for: Frontopolar cortex represents complex features and decision value during choice between environments
Source: Cell Rep. 2023 May 23;42(6):112555. doi: 10.1016/j.celrep.2023.112555 (PMC10320831; doi:10.1016/j.celrep.2023.112555)
Supplement: Document S1. Figures S1–S6 and Tables S1 and S2 [file mmc1.pdf]

Cell Reports, Volume *42*

Supplemental information

Frontopolar cortex represents complex features and decision value during choice between environments

Chun-Kit Law, Nils Kolling, Chetwyn C.H. Chan, and Bolton K.H. Chau

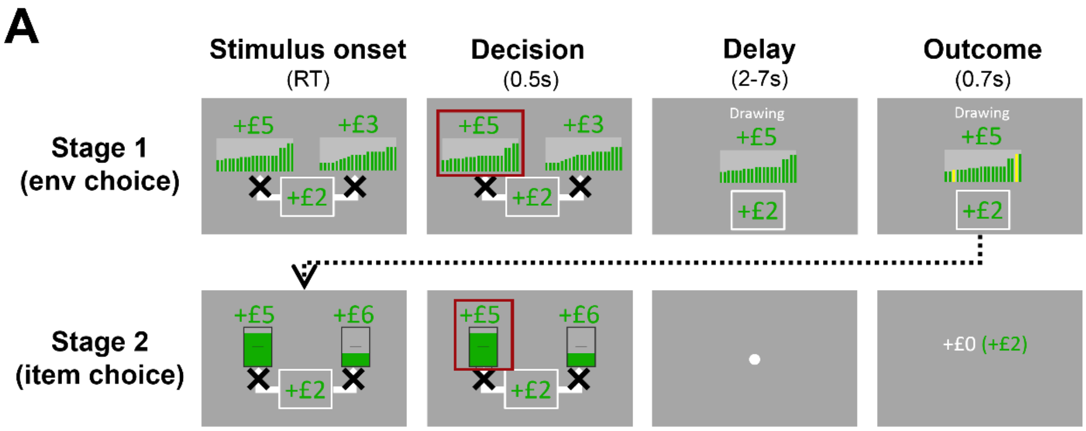

B

	Left environment		Right environment	
	Maximum	Minimum	Maximum	Minimum
Rew mag	3.5	-3.1	3.6	-3
Prob mean	0.693	0.352	0.674	0.321
Prob var	0.075	0.006	0.077	0.008
Prob skew	1.921	-1.822	1.743	-2.034

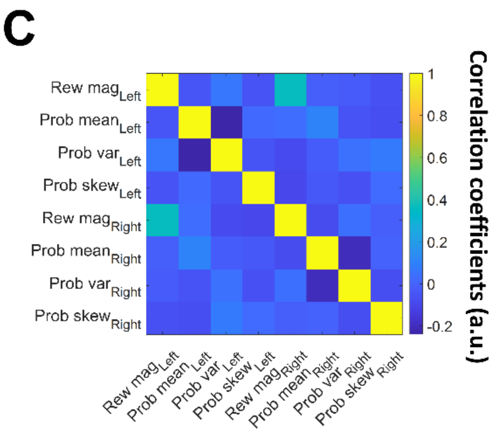

Figure S1. Task structures, Related to Figure 1 and STAR Methods. (A) An example trial of the Unlinked Condition. Figure 1B shows an example trial of the Linked Condition, which was indicated by a pair of arrows between the bonus and the options. The Unlinked Condition had the same structure as the Linked Condition except that it was indicated by crosses on the arrows. **(B)** Descriptive statistics of the environments. **(C)** Correlation matrix of the environments' attributes. Rew mag=reward magnitude of the environment; Prob mean=mean of the reward probabilities of the environment; Prob var=variance of the reward probabilities of the environment; Prob skew=skewness of the reward probabilities of the environment.

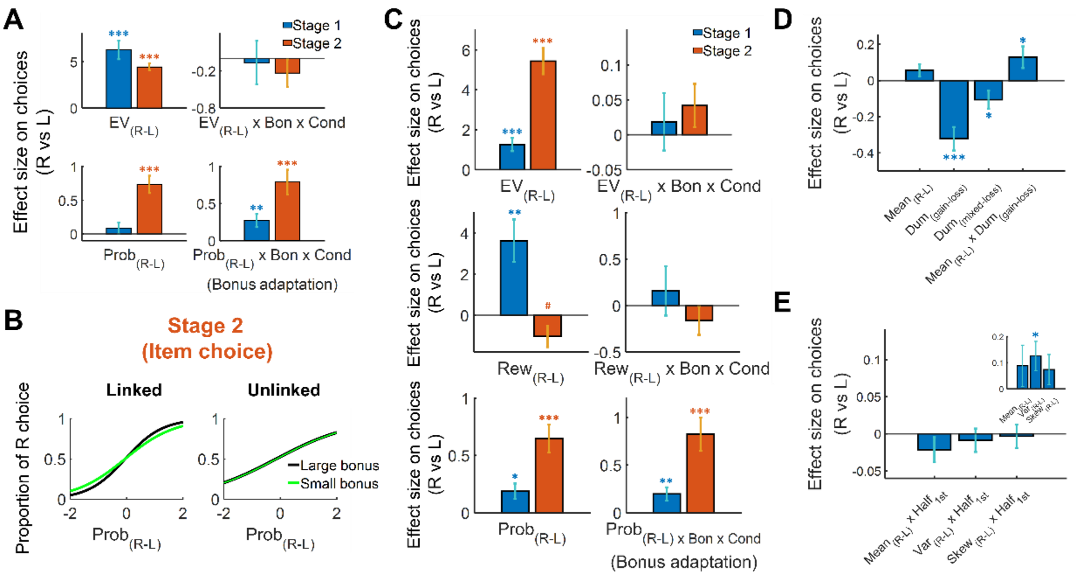

Figure S2. Behavioural results showed that participants understood the task and behaved accordingly, Related to Figure 2. (A) By design, the bonus acquisition depended on the reward probability only. The results of GLM1 in Figure 2A showed that it was the case – there was a presence of three way interaction of Prob$_{(R-L)}$×Bon×Cond (i.e. the bonus adaptation; bottom right panel) but an absence of three way interaction of EV$_{(R-L)}$×Bon×Cond (top right panel) in both Stage 1 environment choice and Stage 2 item choice. **(B)** Similar to Figure 2B, psychometric curves show that during item choice, in Linked Condition the preference for items with larger reward probabilities was stronger when the bonus value became larger (left panel). In contrast, in Unlinked Condition, the preference for larger reward probabilities was independent of the bonus value (right panel). **(C)** An alternative lasso regression to GLM1 was performed to test whether participants made decisions with the essential choice information. It involved the same regressors as in GLM1 but additionally included Rew$_{(R-L)}$ and Rew$_{(R-L)}$×Bon×Cond. Consistent to GLM1, the results also revealed that participants chose according to the options' EVs and showed a Bonus adaptation in both stages. There was also a significant effect of Rew$_{(R-L)}$ in Stage 1 but not in Stage 2, in which Rew$_{(R-L)}$ in Stage 2 could have been fully captured by the EV$_{(R-L)}$ term. **(D)** Figure 2C (GLM2) shows that there was an absence of preference for larger mean reward probability. Since the task involved environments in the gain and loss domains, participants should have stronger preferences for large probabilities in the gain domain and weaker preferences for large probabilities in the loss domain. The difference in preferences should have cancelled out each other in GLM2. To reveal this change in probability preference, first, participants' choices were regressed against the difference in reward magnitude. Second, the resulting residuals were regressed against the following regressors that were related to the reward probability

and the gain/loss condition: (1) $Mean_{(R\text{-}L)}$; (2) $Var_{(R\text{-}L)}$; (3) $Skew_{(R\text{-}L)}$; (4) $Dum_{(gain\text{-}loss)}$ (a dummy variable; 1=trials on which both options involved gains); (5) $Dum_{(mixed\text{-}loss)}$ (a dummy variable; 1=trials on which one option involved gain and one option involved loss; (6) $Mean_{(R\text{-}L)} \times Dum_{(gain\text{-}loss)}$ (an interaction term). We also focused our analysis on the Unlinked trials such that the influence of the bonus could be ruled out. The results showed that the participants did have a stronger preference for larger mean reward probability in gain condition by showing a positive $Mean_{(R\text{-}L)} \times Dum_{(gain\text{-}loss)}$ effect (β=0.129, t_{23}=2.157, P=0.042). **(E)** GLM2 showed that participants had a preference for environments with larger variances (inset). An additional analysis was conducted to test whether the preference for larger variances was confounded by any exploratory behaviour due to unfamiliarity of the task. First, the same GLM2 was performed and the resulting residuals were extracted. Second, the residuals were regressed against $Half_{1st}$ (a dummy variable; first half of trials=1, second half of trials=0) and its interaction terms with preferences of mean, variance, and skewness. The results showed that the preference for environments with larger variances remained comparable throughout the task ($Var_{(R\text{-}L)} \times Half_{1st}$: β=-0.009, t_{23}=-0.556, P=0.584). $EV_{(R\text{-}L)}$=difference in expected value; $Rew_{(R\text{-}L)}$=difference in reward magnitude; $Prob_{(R\text{-}L)}$=difference in reward probability; Bon=bonus value; Cond=Bonus Condition. $Mean_{(R\text{-}L)}$=difference in mean of within-environment probabilities; $Var_{(R\text{-}L)}$= difference in variance of within-environment probabilities; $Skew_{(R\text{-}L)}$=difference in mean of within-environment probabilities; *** denotes P<0.001, ** denotes P<0.01, and * denotes P<0.05 (n=24; one-sample t-test). Error bars represent mean ± SEM.

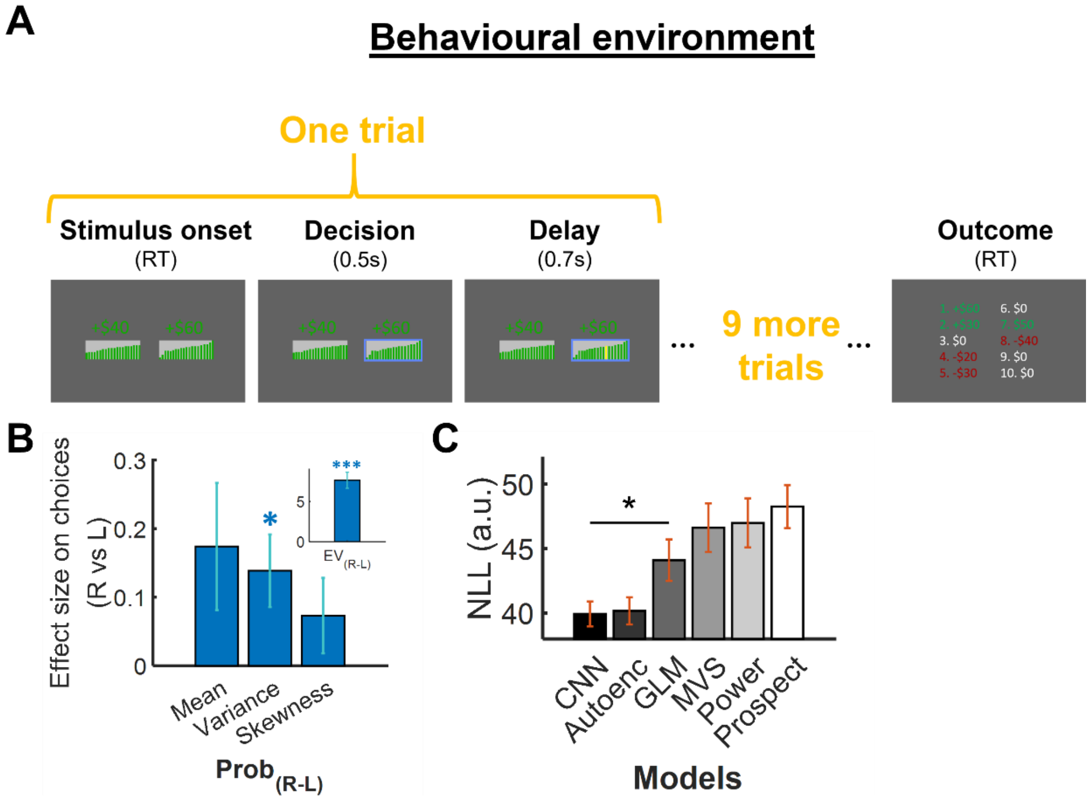

Figure S3. Behavioural results of a simplified behavioural environment choice experiment, Related to Figures 2 and 3. (A) Schematic of the task. On each trial, participants chose between two environments (*Stimulus onset*). After decision, a blue frame surrounded the chosen environment (*Decision*). An item was randomly drawn from the chosen environment and highlighted in yellow after a delay (*Delay*). Each block contained ten trials. Decision outcomes of all chosen items were displayed at the end of the block (*Outcome*). **(B)** Participants chose environments with larger EVs (β=7.641, t_{19}=6.989, P=1.172×10^{-6}) and variances (β=0.139, t_{19}=2.393, P=0.027), which were similar to the behavioural findings in the main experiment that applied the same GLM2 (Fig.2C). *** denotes P<0.001 and * denotes P<0.05 (n=20; one-sample t-test). (**C)** As in Figure 3B, a model comparison showed that CNN outperforms other alternative models in predicting environment choice behaviour (ts$_{38}$<-2.221, Ps<0.033), except the autoencoder (t_{38}=-0.170, P=0.866). * denotes P<0.05 (n=20; independent samples t-test). CNN=convolutional neural network; Autoenc=autoencoder; GLM=general linear model; MVS=mean-variance-skewness model; Power=power law model; Prospect=cumulative prospect theory. Error bars represent mean ± SEM.

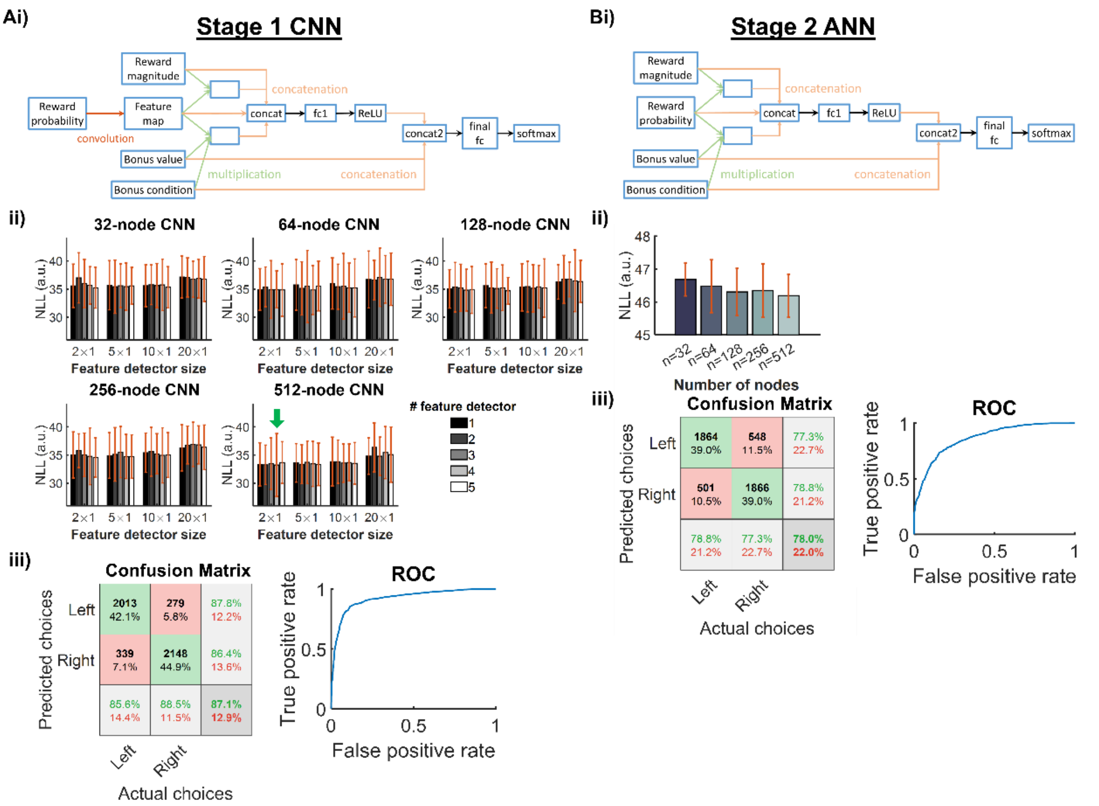

Figure S4. Architectures of the deep learning neural networks for modeling environment and item choices, Related to Figure 3. (A)(i) Full schematic of the CNN shown in Figure 3A. **(ii)** The performance of CNN with different architectures. To optimize the CNN, we systematically varied (1) the size of feature detectors, (2) the number of feature detectors, and (3) the number of nodes involved in fully-connected layer. The CNN with four feature detectors in the size of 2×1 and 512 nodes best predicts the environment choice behaviour (NLL=33.235; green arrow) and thus it was selected for subsequent analyses. **(iii)** Model performance of the best CNN in terms of choice prediction (left panel) and receiver operating characteristic (right panel). **(B)(i)** Full schematic of the artificial neural network (ANN) for Stage 2 item choice data. The Stage 2 ANN is highly similar to the Stage 1 CNN, except without the convolution. **(ii)** Similarly, ANN was optimized by varying the number of nodes in fully-connected layer. The ANN with 512 nodes best predicts the item choice behaviour (NLL=46.195) and thus it was selected for subsequent analyses. **(iii)** Model performance of the best ANN in terms of choice prediction (left panel) and receiver operating characteristic (right panel). NLL=negative log-likelihood. Error bars represent mean ± SEM.

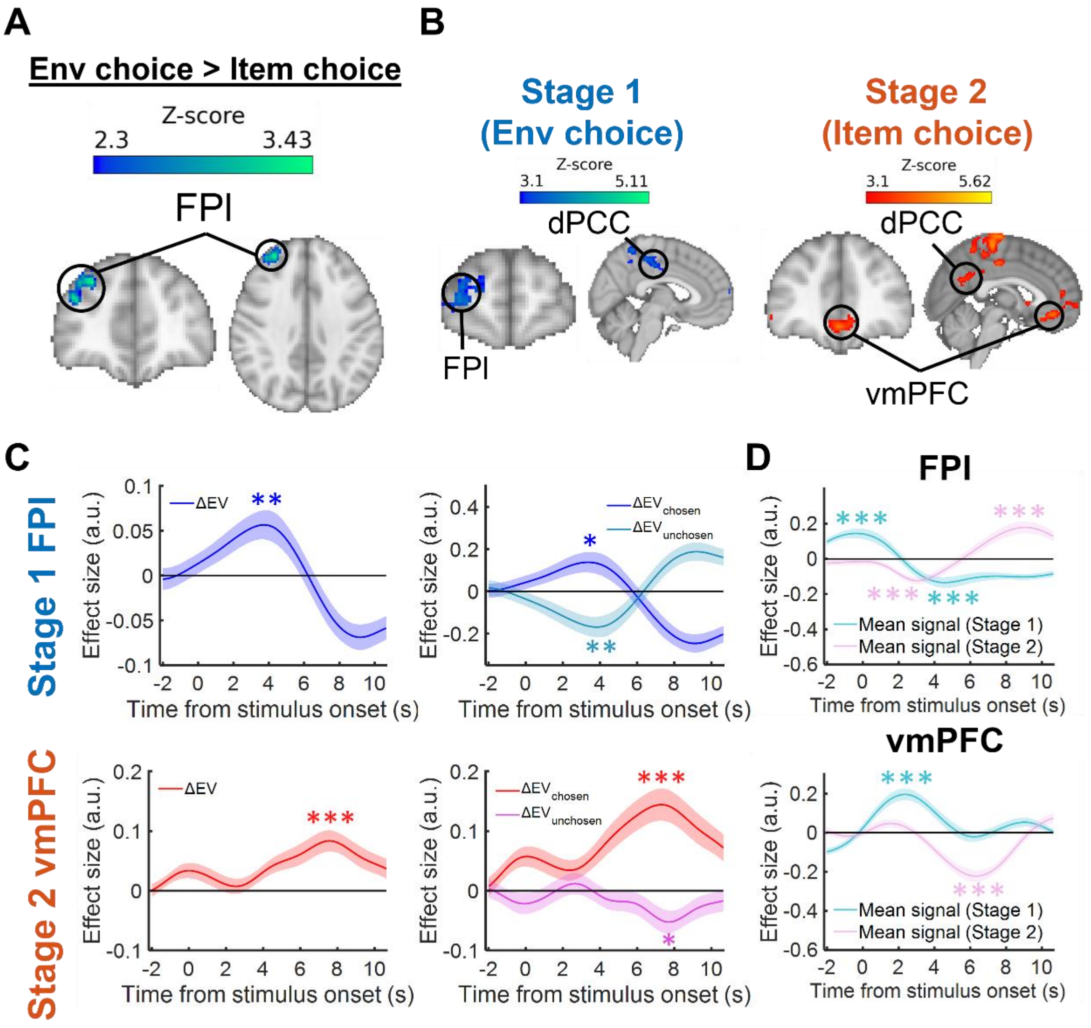

Figure S5. Additional whole-brain contrasts, ROI analyses, and deactivation patterns indicating the dissociable roles of FPl and vmPFC in environment choice and item choice respectively, Related to Figure 4. (A) In Figure 4, we have already observed a double dissociation of ΔDV_{Env} and ΔDV_{Item} signals in FPl and vmFPC, using a cluster-based threshold that requires a cluster to show strong effect but lenient about the cluster size (i.e. $Z>3.1$). Here, an additional whole-brain contrast based on the results in Figure 4 was performed to directly compare these FPl and vmPFC signals. Another commonly used cluster-based threshold of $Z>2.3$ that is more stringent about the cluster size but requires a relatively weaker effect was applied. The results showed that the ΔDV_{Env} signal in Stage 1 was significantly stronger than the ΔDV_{Item} signal in Stage 2 in the FPl (MNI=[28, 44, 36], cluster-based thresholding $Z>2.3$, $P=0.021$). **(B)** Similar results to Figure 4A were obtained when the ΔDV terms in GLM3 was replaced by difference in expected value (ΔEV) terms. Note that the EVs in Linked and Unlinked Conditions involved different computations. In Linked Condition: EV=reward magnitude×reward probability + reward probability×bonus value. In Unlinked Condition: EV=reward magnitude×reward probability. FPl and

vmPFC were identified during environment choice and item choice respectively (FPl: MNI=[38, 52, 24], cluster-based thresholding Z>3.1, P=2.65×10^{-5}; left panel; vmPFC: MNI=[2, 40, -12], cluster-based thresholding Z>3.1, P=1.5×10^{-12}; right panel). While dPCC was also identified in both environment choice (MNI=[10, -18, 38], cluster-based thresholding Z>3.1, P=7.59×10^{-9}; left panel) and item choice (MNI=[8, -52, 36], cluster-based thresholding Z>3.1, P=1.16×10^{-18}; right panel). **(C)** In addition to ΔEV signals, EV signals related to the chosen and/or unchosen item are also commonly found in vmPFC. Consistent to previous findings, we showed that vmPFC activity did not only correlated with ΔEV_{Item} (β=0.082, t_{23}=4.628, P=1.18×10^{-4}, BF_{10}=234.168; bottom left panel), but also $EV_{chosen\ item}$ (β=0.136, t_{23}=5.076, P=3.87×10^{-5}, BF_{10}=636.383; bottom right panel, red line) and $EV_{unchosen\ item}$ (β=-0.051, t_{23}=-2.806, P=0.010, BF_{10}=4.831; bottom right panel, magenta line). The same analyses were applied to FPl. Similarly, FPl activity correlated with ΔEV_{Env} (β=0.054, t_{23}=3.422, P=0.002, BF_{10}=16.774; top left panel), as well as $EV_{chosen\ env}$ (β=0.129, t_{23}=2.788, P=0.010, BF_{10}=4.668; top right panel, blue line) and $EV_{unchosen\ env}$ (β=-0.161, t_{23}=-3.434, P=0.002, BF_{10}=17.207; top right panel, cyan line). **(D)** Both FPl and vmPFC are parts of the default mode network, which are supposed to show overall deactivations when they are engaged in a task.[1,2] Despite its functional significance is less clear in the literature, the overall deactivation can be used as an indicator of whether these regions are engaged in certain choices. We examined the mean signals of the FPl and vmPFC which provided further evidence of their dissociable roles in environment choice and item choice. For the FPl, during environment choice a deactivation (i.e. engaged) was observed at a time point similar to the ΔDV_{Env} signal as shown in Figure 4Bi (at 4.840s; β=-0.182, t_{23}=-5.874, P=5.491×10^{-6}, BF_{10}=3.752×10^{3}; top panel, cyan line), although prior to that the mean signal also ramped up transiently before stimulus onset (β=0.172, t_{23}=6.770, P=6.633×10^{-7}, BF_{10}=2.613×10^{4}; top panel, cyan line). In contrast, during item choice there was an opposite pattern – after an early overall deactivation (β=-0.125, t_{23}=-6.183, P=2.623×10^{-6}, BF_{10}=7.380×10^{3}; top panel, magenta line), the overall FPl activity ramped up (i.e. disengaged) after about 5s (β=0.232, t_{23}=6.526, P=1.169×10^{-6}, BF_{10}=1.551×10^{4}; top panel, magenta line). Although it is less clear why there was a mix of mean signal activation and deactivation, it is obvious that in the FPl the mean signal showed opposite patterns during environment choice and item choice. On the contrary, the vmPFC mean signal exhibited a deactivation (i.e. engaged) during item choice (β=-0.218, t_{23}=-7.423, P=1.512×10^{-7}, BF_{10}=1.026×10^{5}; bottom panel, magenta line), but an activation (i.e. disengaged) during environment choice (β=0.190, t_{23}=6.272, P=2.123×10^{-6}, BF_{10}=8.957×10^{3}; bottom panel, cyan line). *** denotes

P<0.001 and * denotes *P*<0.05 (n=24; one-sample t-test). Shading represent mean ± SEM.

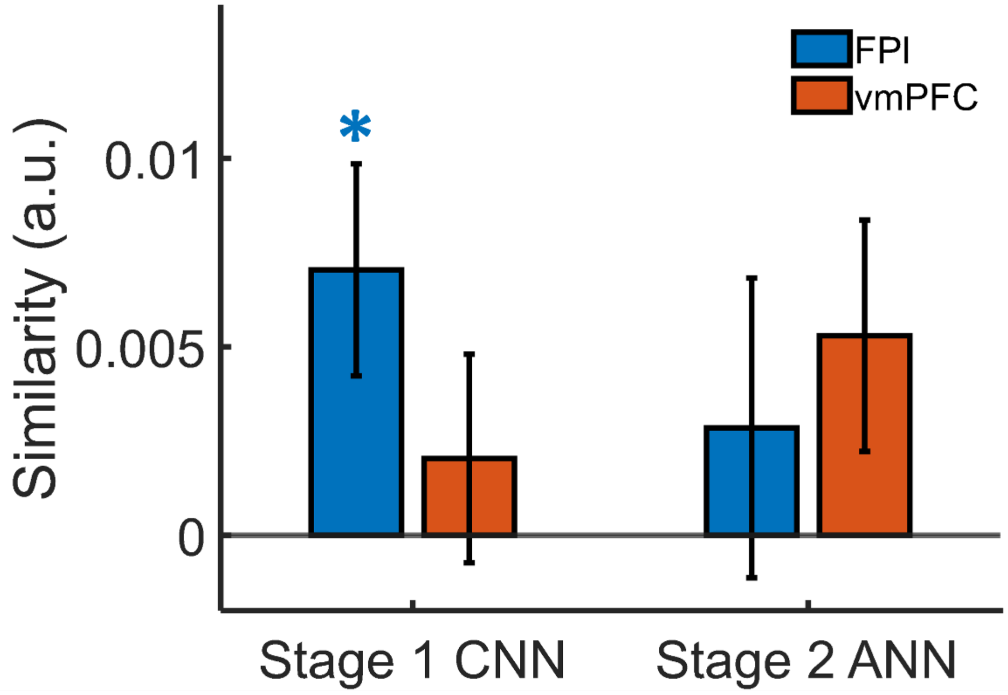

Figure S6. Environment-choice-like CNN and item-choice-like ANN shared similar computational representations with FPl and vmPFC respectively, Related to Figure 7. The RSA results in Figure 7 have already showed that Stage 1 CNN (environment-choice-like) shared similar representations only to FPl and not vmPFC. Here, we further tested whether Stage 2 ANN (item-choice-like) showed the opposite patterns. As expected, Stage 2 ANN was not similar to FPl (*rho*=0.002, signed-rank *P*=0.954, permutation *P*=0.276). Also, a marginally significant representational similarity was observed between the Stage 2 ANN and vmPFC (*rho*=0.005, signed-rank *P*=0.092, permutation *P*=0.072). * denotes $P<0.05$ (n=24). Error bars represent mean ± SEM.

Table S1. Descriptive statistics of GLM1, Related to Figure 2.

	β	*SEM*	*t*	*df*	*p*
**Stage 1**					
Intercept	0.570	0.164	3.471	23	0.002
$EV_{(R-L)}$	6.264	1.011	6.196	23	2.543×10^{-6}
$Prob_{(R-L)}$	0.086	0.084	1.029	23	0.314
Bon	0.054	0.057	0.944	23	0.355
Cond	-0.333	0.105	-3.183	23	0.004
$EV_{(R-L)}$×Bon×Cond	-0.067	0.352	-0.191	23	0.850
$Prob_{(R-L)}$×Bon×Cond	0.271	0.088	3.065	23	0.006
**Stage 2**					
Intercept	0.084	0.067	1.248	23	0.225
$EV_{(R-L)}$	4.420	0.382	11.582	23	4.468×10^{-11}
$Prob_{(R-L)}$	0.734	0.129	5.704	23	8.291×10^{-6}
Bon	0.027	0.032	0.834	23	0.413
Cond	-0.032	0.092	-0.347	23	0.732
$EV_{(R-L)}$×Bon×Cond	-0.235	0.222	-1.061	23	0.300
$Prob_{(R-L)}$×Bon×Cond	0.790	0.167	4.735	23	9.007×10^{-5}

$EV_{(R-L)}$=difference in expected value; $Prob_{(R-L)}$=difference in reward probability; Bon=bonus value; Cond=Bonus Condition.

Table S2. Whole-brain analysis results, Related to Figure 4

	x	y	z	Max *Z*-score	P-value	#voxels
**ΔDV_{Env}**						
Lateral frontopolar cortex	38	52	22	4.31	1.19×10^{-7}	486
Dorsal posterior cingulate cortex	16	-32	42	4.65	2.44×10^{-13}	1074
Frontal eye field	4	44	30	4.12	0.001	180
Inferior frontal gyrus	56	28	0	4.29	0.035	100
Lateral orbitofrontal cortex	42	26	-18	4.46	0.004	151
Postcentral gyrus	24	-40	66	4.11	0.001	188
Inferior parietal lobule	48	-62	32	5.28	7.09×10^{-29}	3281
Superior temporal gyrus	-62	-30	20	4.63	8.53×10^{-09}	598
Right cerebellum	18	-76	-32	3.9	0.014	122
Left cerebellum	-22	-86	-30	-5	1.22×10^{-13}	780
**ΔDV_{Item}**						
Ventromedial prefrontal cortex	2	40	-10	4.47	7.77×10^{-21}	2055
Dorsal posterior cingulate cortex	8	-52	36	5.26	1.73×10^{-24}	2599
Temporoparietal junction	52	-20	-10	5.99	<0.0001	6932
Supramarginal gyrus	-54	-38	24	4.95	1.22×10^{-18}	1749
Medial frontopolar cortex	-16	62	12	3.61	2.47×10^{-4}	233
Temporal fusiform cortex	-34	-34	-24	3.98	0.014	123
Angular gyrus	-50	-68	28	3.69	0.015	121
Right cerebellum	28	-74	-36	3.77	0.038	99
Left cerebellum	-24	-74	-36	5.11	3.84×10^{-12}	947
**$\Delta DV_{Env} - \Delta DV_{Item}$**[a]						
Lateral frontopolar cortex	28	44	36	3.4	0.021	348

ΔDV_{Env}=decision value difference between environments; ΔDV_{Item}=decision value difference between items.

[a]Cluster-based thresholding $Z>2.3$

Supplemental references

[S1] Buckner, R.L., Andrews-Hanna, J.R., Schacter, D.L., 2008. *The Brain's Default Network*: *Anatomy, Function, and Relevance to Disease*. Ann. N. Y. Acad. Sci. 1124, 1–38. https://doi.org/10.1196/annals.1440.011

[S2] Gusnard, D.A., Raichle, M.E., 2001. Searching for a baseline: Functional imaging and the resting human brain. Nat. Rev. Neurosci. 2, 685–694. https://doi.org/10.1038/35094500
